# A single generation of domestication heritably alters the expression of hundreds of genes

**DOI:** 10.1038/ncomms10676

**Published:** 2016-02-17

**Authors:** Mark R. Christie, Melanie L. Marine, Samuel E. Fox, Rod A. French, Michael S. Blouin

**Affiliations:** 1 Department of Biological Sciences, Purdue University, West Lafayette, 47907-2054 IN, USA; 2 Department of Forestry and Natural Resources, Purdue University, West Lafayette, 47907-2054 IN, USA; 3 Department of Integrative Biology, Oregon State University, Corvallis, 97331-2914 Oregon, USA; 4 Department of Biology, Saint Martin's University, Lacey, 98503-7500 WA, USA; 5 Oregon Department of Fish and Wildlife, The Dalles, 97058-4364 Oregon, USA

## Abstract

The genetic underpinnings associated with the earliest stages of plant and animal domestication have remained elusive. Because a genome-wide response to selection can take many generations, the earliest detectable changes associated with domestication may first manifest as heritable changes to global patterns of gene expression. Here, to test this hypothesis, we measured differential gene expression in the offspring of wild and first-generation hatchery steelhead trout (*Oncorhynchus mykiss*) reared in a common environment. Remarkably, we find that there were 723 genes differentially expressed between the two groups of offspring. Reciprocal crosses reveal that the differentially expressed genes could not be explained by maternal effects or by chance differences in the background levels of gene expression among unrelated families. Gene-enrichment analyses reveal that adaptation to the novel hatchery environment involved responses in wound healing, immunity and metabolism. These findings suggest that the earliest stages of domestication may involve adaptation to highly crowded conditions.

## Abstract (web summary)

Little is known about the genetic underpinnings of the earliest stages of plant and animal domestication. Here, the authors show that steelhead trout respond to a single generation of domestication selection with large, heritable changes in expression of genes regulating immunity and metabolism.

## Graphical abstract

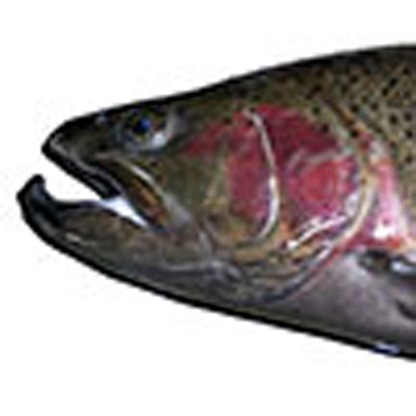

Humans have selected for novel and useful traits in a wide variety of plants and animals. Over thousands of generations, this selection process has resulted in domesticated plants and animals that often bare little resemblance to their ancestors, but that nonetheless fill fundamental roles in the development and functioning of modern societies^1^,^2^. The importance of domestication cannot be understated: agriculture, human population growth and even contemporary infectious diseases are just some of the products of this fundamental evolutionary process^1^,^2^,^3^. Despite the importance of domestication, the underlying genetic mechanisms by which domestication occurs, particularly during incipient stages, remain largely unknown. Recent genome-wide investigations of species that have been domesticated for thousands of years have revealed that a large number of independent genomic regions are responsible for extensive changes in behaviour, colouration, morphology and physiology^4^,^5^,^6^. Investigations into more recently domesticated species, some of which continue to interbreed with their wild progenitors, have identified a comparatively modest number of genomic regions that show signals of past domestication^7^,^8^. This reduced power to detect recent domestication selection is coincident with theoretical work illustrating that even strong selection can only cause modest changes to gene frequencies over several generations^9^,^10^.

Despite the long period of domestication typically required to detect signatures of artificial selection at the genomic level, recent work has revealed that genetic adaptation to captivity can occur exceptionally quickly, sometimes within only a handful of generations^11^,^12^,^13^,^14^. For example, fish reared in hatcheries can show substantial adaptation to captivity after just a single generation of selection^15^,^16^. Such rapid adaptation could occur via three complementary mechanisms: (i) selection could result in small allele frequency changes at many loci, as in traditional quantitative genetics models^17^, (ii) selection could act directly on a few regulatory loci^18^ or (iii) there could be physical changes to the genome that are functionally relevant but that do not involve a change in the nucleotide sequence (that is, heritable epigenetic modifications)^19^. These three mechanisms leave different signatures at the genomic level, but all create changes that can be directly detected by measuring the global patterns of gene expression.

To test whether we could identify incipient domestication at the mRNA transcript level, we compared patterns of gene expression in offspring of first-generation hatchery (that is, hatchery-origin) and wild (that is, wild-origin) steelhead trout collected directly from the Hood River, Oregon. Previous work in this system revealed that first-generation hatchery fish averaged 85% of the lifetime reproductive success of wild fish when spawning in the wild^20^, but nearly twice the lifetime reproductive success of wild fish when spawned in captivity^16^. First-generation hatchery fish had wild-origin parents and only spent their first year in the hatchery before being released into the wild. A series of crosses involving two first-generation hatchery fish (H × H), two wild fish (W × W) or one hatchery and one wild fish (H × W and W × H reciprocal crosses) were performed at the Parkdale hatchery (Supplementary Fig. 1; Supplementary Table 1). The offspring were reared in an identical environment at the hatchery until the swim-up fry stage (yolk sac absorption), at which point the fry were collected for RNA-Seq. If incipient domestication is occurring in this system, then we expected to find two patterns: (i) there should be greater differences in gene expression between the offspring of two first-generation hatchery fish (H × H) and two wild fish (W × W) than between the offspring of an equal number of families having equal hatchery ancestry and (ii) any differences in gene expression should not be solely due to maternal effects, which could be an environmental effect and not necessarily due to domestication selection^21^,^22^.

We find that there are hundreds of genes that are differentially expressed (DE) between the offspring of wild fish (W × W) and of the offspring of hatchery fish (H × H) reared in a common environment. By using reciprocal crosses, we further show that these differences in gene expression cannot be explained as maternal effects, sampling noise, or false discovery. Thus, our data suggest that the very first stages of domestication are characterized by massive, heritable changes to gene expression. That the DE genes were dominated by pathways in wound repair, immunity and metabolism adds to growing evidence that adaptation to crowded conditions is an important early stage of domestication.

## Results

### Main effects

**Figure 1:**
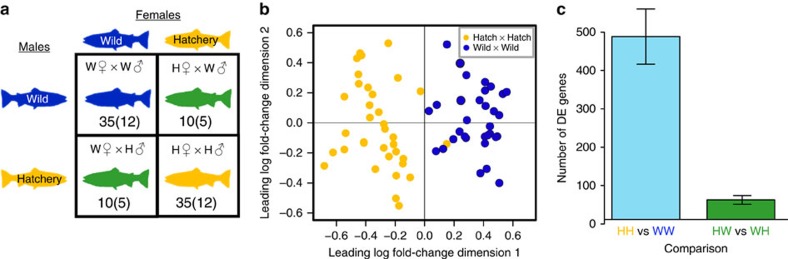
Genome-wide differences in gene expression between the offspring of first-generation hatchery fish and wild fish reared in an identical environment. (**a**) Both first-generation hatchery fish (H) and wild fish (W) were crossed in the hatchery to create offspring with two wild parents (W × W), two hatchery parents (H × H), one hatchery mother and one wild father (H × W), or one wild mother and one hatchery father (W × H). Numbers represent the total number of individuals and families (in parentheses) sequenced in this study. (**b**) Multidimensional scaling plot illustrating the differences in the gene expression profiles for the offspring of first-generation hatchery fish (H × H; yellow circles) and the offspring of wild fish (W × W; blue circles). A total of 723 genes were differentially expressed between the two groups. (**c**) To compare the main effect (H × H vs W × W) with background rates of differential expression expected between two groups of families having equal hatchery backgrounds, we compared the number of genes differentially expressed between H × W and W × H fish vs H × H and W × W fish from the 5 matrices in which all cross types were sequenced. There were 426 more differentially expressed genes in the H × H vs W × W comparison than in the H × W vs W × H comparison. To account for siblings we randomly sampled one offspring per family, calculated the number of DE genes, repeated the process 100 times and calculated the mean and 95% confidence intervals (illustrated with error bars; 40 individuals total, 2 siblings per family). This result also suggests that the difference between H × H and W × W fish is a heritable genetic effect rather than simply a maternal effect of the juvenile environment experienced by the mother.

Remarkably, we found that there were 723 genes DE between the offspring of wild fish (W × W) and the offspring of first-generation hatchery fish (H × H), where the offspring represent 70 individuals from each of the 24 unrelated families (1–4 individuals sequenced per family; Fig. 1b; Supplementary Data 1). If siblings were randomly removed such that a single individual represented each family, we still detected an average of 579 DE genes (95% confidence interval=390–770; Supplementary Fig. 2). Out of the 723 DE genes, substantially more genes were upregulated in the offspring of the hatchery fish in comparison with the offspring of wild fish (458 vs 265, *χ*^2^=50.18; *P*<0.001) perhaps because the common environment was a hatchery. In addition to the 12 H × H and 12 W × W families, we also created five 2 × 2 matrices by crossing a wild and a hatchery male factorially with a wild and a hatchery female, respectively (Fig. 1a; Supplementary Table 1). This design allowed us to independently examine the background number of DE genes expressed between the two groups after controlling for their hatchery background (that is, we compared the number of DE genes between H × W and W × H offspring with the number of DE genes between H × H and W × W offspring). To avoid comparing different numbers of individuals, we only used W × W and H × H offspring from the same matrices as the W × H and H × W offspring. We could only detect an average of 51 genes DE between the H × W and W × H offspring in comparison with an average of 477 genes detected between the H × H and W × W offspring (Fig. 1c). This result clearly illustrates that there are differences in gene expression between the offspring of hatchery and the offspring of wild fish that are substantially beyond the level expected between two groups of unrelated families having equivalent amounts of hatchery ancestry (that is, the results cannot be explained by sampling noise or false discovery).

### Maternal effects

**Figure 2:**
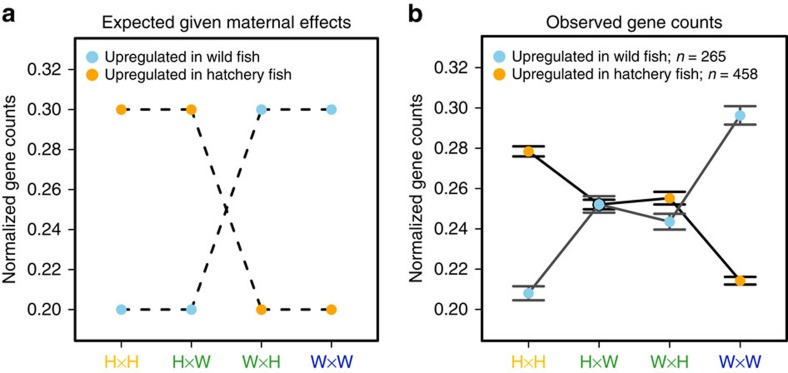
Examination of maternal effects using reciprocal crosses. Here “upregulated” means more highly expressed in that group. (**a**) Expected pattern of DE genes for purely maternal effects. The offspring of H × H and H × W crosses shared a mother. The offspring of the W × H and W × W crosses also shared a mother (See Fig. 1). If there were maternal effects, we would expect, after normalizing counts across all DE genes, that the offspring of H × W crosses would be more similar to the H × H offspring and that the offspring of the W × H fish would be more similar to the W × W fish. (**b**) We observed that most of the genes have an additive effect, in that expression values for H × W and W × H offspring are intermediate. Bars represent 95% confidence intervals for all DE genes (n=723). This result suggests that very few genes are DE due to maternal effects.

If the differences in gene expression were simply due to maternal effects (the mothers of the hatchery offspring and the wild offspring experienced different environments), then we would expect to see two patterns. First, the number of DE genes between H × H and W × W should equal that between H × W and W × H. This was clearly not the case (Fig. 1c). Second, normalized gene counts should be similar between the offspring of H × H fish and the offspring of H × W fish because both groups of offspring shared the same hatchery mother (Fig. 2a). Likewise we would expect to see normalized gene counts that were similar between the offspring of W × W fish and W × H fish because both groups shared the same wild mother^23^. However, across all matrices, we observed nearly additive effects, where the normalized gene counts for both H × W and W × H fish were intermediate between the gene counts for the H × H and W × W offspring (Fig. 2b). This result strongly suggests that the differences in gene expression between the H × H and W × W offspring are not due to the different environments experienced by their mothers. Although one can find a handful of genes consistent with purely maternal effects (Supplementary Fig. 3), the vast majority of DE genes did not show this pattern.

### Genetic drift

Using single-nucleotide polymorphisms called after aligning all RNA-Seq data to the *O. mykiss* genome^24^, we calculated a genome-wide *F*_*ST*_ between the H × H and W × W offspring equal to 0.009. This low level of divergence is of a magnitude similar to what one would expect from sampling error alone when comparing *F*_*ST*_ between two similarly sized samples^25^ and suggests that genetic drift is an unlikely explanation for the differences in expression between the offspring of hatchery and wild parents. If genetic drift were driving the differences in gene expression between the H × H and W × W offspring, we would expect to see much larger genome-wide levels of divergence. Second, the genome-wide *F*_*ST*_ between the H × H vs W × W offspring was equivalent to *F*_*ST*_ between the W × H vs H × W offspring (0.009 vs 0.0088, respectively). This observation suggests that offspring with identical amounts of hatchery ancestry have the same level of divergence as offspring with different amounts of hatchery ancestry. If genetic drift were responsible for the differences in gene expression illustrated in Fig. 1c, then we would expect *F*_*ST*_ between the H × H and W × W offspring to be substantially greater than *F*_*ST*_ between the W × H and H × W offspring, which we did not observe. The large extent of divergence that occurs at the gene-expression level, but not at the genomic level, suggests that selection and not genetic drift is responsible for the large differences in expression detected between the offspring of wild and first-generation hatchery fish.

### Gene location and gene enrichment

Where in the genome are the DE genes located? We next aligned the contigs associated with the DE genes against the *O. mykiss* genome^24^ and found a positive relationship between the number of DE genes and chromosome size (Supplementary Fig. 4). This relationship suggests that the DE genes involved in responding to the captive environment are distributed proportionately throughout the genome rather than clustered on a single or small number of chromosomes.

We next performed a gene-enrichment analysis on the DE genes, which can be useful for identifying the traits that may be under selection in the captive environment. We identified a large number of genes that are associated with three general categories: coagulation/wound healing, immune response and metabolism (Table 1). Because fish from hatcheries often have abraded fins and other injuries^26^, it is possible that hatchery fish respond to selection by upregulating genes to prevent or repair the increased wounding that can occur in the captive environment. Furthermore, it is well-known that the aggressive tendencies of steelhead, along with the ancestors of many domesticated animals, are exacerbated when the animals are reared at high densities. These behavioural traits can translate into injury via increased agonistic encounters^27^,^28^. Previous work from this and other systems suggests that density can play a critical role in facilitating rates of genetic adaptation to captivity^16^,^29^,^30^. The fact that hatchery fish are reared in closed confines and at high densities relative to wild fish may also play a role in immune-related processes (Table 1), where diseases are known to increase in prevalence in crowded vs uncrowded conditions. Taken together, these results suggest that rearing density may play an important role in facilitating genetic adaptation to captivity, and that adjusting to large numbers of conspecifics may be an important first step towards domestication.

## Discussion

*O. mykiss* are one of the few fish species considered to have been fully domesticated^31^. Phenotypic responses to selection routinely occur in this species with less than ten generations of captive breeding. However, this is the first study to demonstrate that the earliest stages of domestication are characterized by large changes in heritable patterns of gene expression. As subsequent generations of domestication accrue, we speculate that the regulatory changes to expression become codified with gradual and more targeted shifts in allele frequencies (for example, selective sweeps). We hypothesize that adaptation to crowded conditions may drive much of this early domestication. Regardless of the mechanism, it is remarkable that a single generation of domestication can translate into heritable differences in expression at hundreds of genes.

## Methods

### Experimental design

Crosses were performed with winter-run steelhead from the Hood River, Oregon, which are listed as threatened under the Endangered Species Act^32^. All steelhead returning to spawning grounds in the Hood River were first passed over the Powerdale dam, which was a complete barrier to migrating fishes. Every fish passed over the dam was individually handled, and samples of scales and fin tissue were collected for aging and genetic analysis by staff of the Oregon Department of Fish and Wildlife. Also recorded were the length, weight, sex and run timing of every fish (none of these traits differed between hatchery and wild fish). First-generation hatchery fish have been released since 1992 as part of programme to increase the local abundance of the steelhead population^11^,^15^,^33^. Steelhead were easily categorized as hatchery or wild origin because all hatchery fish had their adipose fin clipped before being released as juveniles. To create first-generation hatchery fish, wild adult steelhead were collected at the Powerdale dam and spawned at Parkdale fish hatchery. The hatchery fish were reared in the hatchery environment for 1 year, after which they were released into the Hood River near the spawning sites of wild fish. Both the first-generation hatchery fish and wild-born steelhead subsequently migrated downriver to the ocean, where they matured, on average, for 3 additional years before returning to the Hood River to spawn (or be collected for the crosses used in this experiment; see Supplementary Fig. 1). The first-generation hatchery parents used in this study came from broodyears 2006 and 2007. In broodyear 2006 a total of 17,061 fish were released and in broodyear 2007 a total of 26,094 fish were released. The dimension of the final rearing tank was 6,500 cubic feet, which translates to 2.62 and 3.85 fish per cubic foot of water in 2006 and 2007, respectively (or 0.093 and 0.14 fish per liter, respectively).

In 2010, upon returning to the Hood River as adults, both wild-born and first-generation hatchery fish were collected and crosses were performed in the hatchery. We created twelve 2 × 2 matrices by crossing a hatchery (H) and a wild (W) male factorially with a hatchery and a wild female (Fig. 1a; Supplementary Table 1). We initially sequenced two male and two female offspring per family from only the H × H and W × W families from six matrices (that is, six independent families of each type, *n*=48). There were very few DE genes between male and female offspring (sex identified via PCR of a sex-specific marker OmyY1 ref. 34; Supplementary Fig. 2), so for subsequent sequencing we used only female offspring. To test for maternal effects, we next sequenced two females per family from all four cross types (W × W, W × H, H × W, H × H) from an additional five matrices. We also added one more offspring each from an additional W × W and H × H cross for a total of 90 individuals sequenced (Supplementary Table 1). The offspring of each cross (that is, each family) were reared under identical conditions such that the only difference between the offspring was their parentage (that is, whether their parents were born in the hatchery or the wild; see Supplementary Fig. 1). All offspring were reared until the swim-up fry stage at which point the fry were frozen in liquid nitrogen and transferred to a −80 °C freezer for storage. All fry were collected on the same day and at the same time to minimize changes in gene expression due to development or circadian rhythms^35^. No obvious circadian genes were identified among the DE genes (Supplementary Data 1).

### RNA-seq

Total RNA was isolated from frozen fry using a modified trizol/chloroform protocol described previously^36^. RNA was extracted using Trizol Reagent (Invitrogen). Total RNA was treated for 10 min at 65 °C with RNAsecure reagent (Ambion) and for 10 min at 37 °C with RNase-free TURBO DNase (Ambion). Total RNA was further purified using RNAeasy Mini Kit (Qiagen) according to the manufacturer's protocol. Concentration, integrity and extent of contamination by ribosomal RNA were assessed using Qubit Fluorometer (Life Technologies) and Bioanalyzer 2100 (Agilent Technologies). Preparation of cDNA for Illumina Genome Analyzer 2500 was completed following the TruSeq RNA Sample Preparation Kit v2 protocol (Illumina). Each individual had a unique barcode and barcodes were assigned evenly across sample type. Individuals were randomly allocated across lanes, and each lane had 10–12 individuals. This procedure resulted in an average of 20 million 100-bp single-end reads for each individual, with a total of 1.8 billion reads across the entire study.

### Main effects

We first processed the reads by replacing any nucleotides with a quality score of 20 or lower with an ‘*N'*. We next used Bowtie2 (ref. 37) using the ‘very-sensitive' option to align all reads to the population-specific transcriptome^38^. Because there are a large number of homeologs within the *O. mykiss* genome^24^, we used strict criteria for read alignment where each read was only allowed to match to a single contig and reads that matched at multiple locations were discarded. After filtering and alignment, we were left with 956 million reads. For each SAM file, we counted the number of alignments to each contig for each individual. We next used the program edgeR^39^ to test for differential gene expression between H × H and W × W individuals. For each gene, we required all individuals to have minimum of 10 counts per million (c.p.m.) resulting in a minimum of 100 counts per gene per individual and average number of 200 counts per gene per individual given our average coverage of 20 million reads (filtering at a less stringent threshold of 1 c.p.m. gave almost identical results, with only 9 different genes changing in statistical significance). There were a considerable number of DE genes identified between each H × H and each W × W family (that is, family-level effects), thus we used a generalized linear model to control for differences between families^40^. All genes with an FDR-adjusted *P* value≤0.05 (as implemented in edgeR) were counted as DE. We calculated the number of DE genes using all H × H and W × W offspring (Fig. 1) and after accounting for siblings. To account for siblings, we: (i) randomly sampled one offspring per family, (ii) calculated the number of DE genes, (iii) repeated the process 100 times and calculated the mean and 95% confidence intervals (Supplementary Fig. 2).

To test whether differences in gene expression between the offspring of hatchery and the offspring of wild fish were substantially above the level expected between two groups of unrelated individuals having equivalent amounts of hatchery ancestry, we used the same approach outlined above to test for DE genes between H × W vs W × H individuals (Fig. 1c). Because there were fewer H × W and W × H individuals compared with H × H and W × W individuals, we only used H × H and W × W individuals from the same matrices for comparison. To account for siblings, we used the same random sampling process described above. Using even more stringent criteria for accepting DE genes by requiring a minimum fold change≥1.5 in addition to the FDR-adjusted *P* value≤0.05 resulted in 28% fewer DE genes overall, but the same qualitative patterns remained. When using these more stringent criteria, the mean fold-change values were substantially larger in the comparison of H × H and W × W offspring than for the comparisons between H × W and W × H offspring (*P*<0.009) in addition to the number of DE genes.

### Testing for maternal effects

All counts were normalized across all genes and each DE gene was classified as upregulated in wild fish or upregulated in hatchery fish based upon the back-transformed values of the associated log fold change.

### Gene enrichment

To identify DE genes, we annotated the entire steelhead transcriptome via blastx to the swiss-prot and uni-prot databases, resulting in the identification of 15,763 genes. All DE genes were found to have significant homology with known genes (largest *E* value=7 × 10^−5^), though the functions associated with many of these genes remain uncharacterized. The transcriptome sequence names associated with each of the DE genes and their corresponding *E* value obtained from the blastx search are available in Supplementary Data 2 (and the raw sequences are available in Supplementary Data 3). We next used blast2go to identify gene ontology terms associated with each annotated contig from the transcriptome^41^, where 41% of all contigs were determined to have significantly associated GO terms when using the annotated transcriptome as the reference. Lastly, we performed gene-enrichment analysis to identify GO terms that were over-represented with respect to the list of all GO terms associated with the DE genes^42^.

### Code availability

The R Script used for performing tests of differential gene expression is available as Supplementary Software 1. Both R and edgeR are freely distributed at https://cran.r-project.org/ and https://www.bioconductor.org/, respectively.

## Additional information

**Accession Codes:** the RNA-seq data generated in this study have been deposited in the NCBI Sequence Read Archive under SRP study accession number SRP067742.

**How to cite this article:** Christie, M. R. *et al*. A single generation of domestication heritably alters the expression of hundreds of genes. *Nat. Commun.* 7:10676 doi: 10.1038/ncomms10676 (2016).

## Supplementary Material

Supplementary Information Supplementary Figures 1-4 and Supplementary Table 1

Supplementary Data 1 Differentially expressed genes that were identified between the offspring of wild born fish (WxW) and the offspring of first-generation hatchery fish (HxH). Genes are sorted by log fold change (log FC). Also reported are the standardized protein names, full gene names, and false discovery rate adjusted p-value (FDR) for tests of differential expression.

Supplementary Data 2 List of the contig names associated with the 723 genes that were identified as differentially expressed between the offspring of wild born fish (WxW) and the offspring of first-generation hatchery fish (HxH). Also reported are the standardized protein names and the E-value associated with the blastx homology search. Contigs are presented in the same order as Supplementary Data 1. Sequence data for each listed contig can be found in Supplementary Data 3 and the entire steelhead transcriptome can be found at: http://salmon.cgrb.oregonstate.edu/

Supplementary Data 3 File containing the FASTA sequences of the contigs associated with the 723 genes that were identified as differentially expressed between the offspring of wild born fish (WxW) and the offspring of first-generation hatchery fish (HxH).

Supplementary Software 1 R script used to invoke the program ‘edgeR' to perform tests of differential gene expression.

## Figures and Tables

**Table 1:**
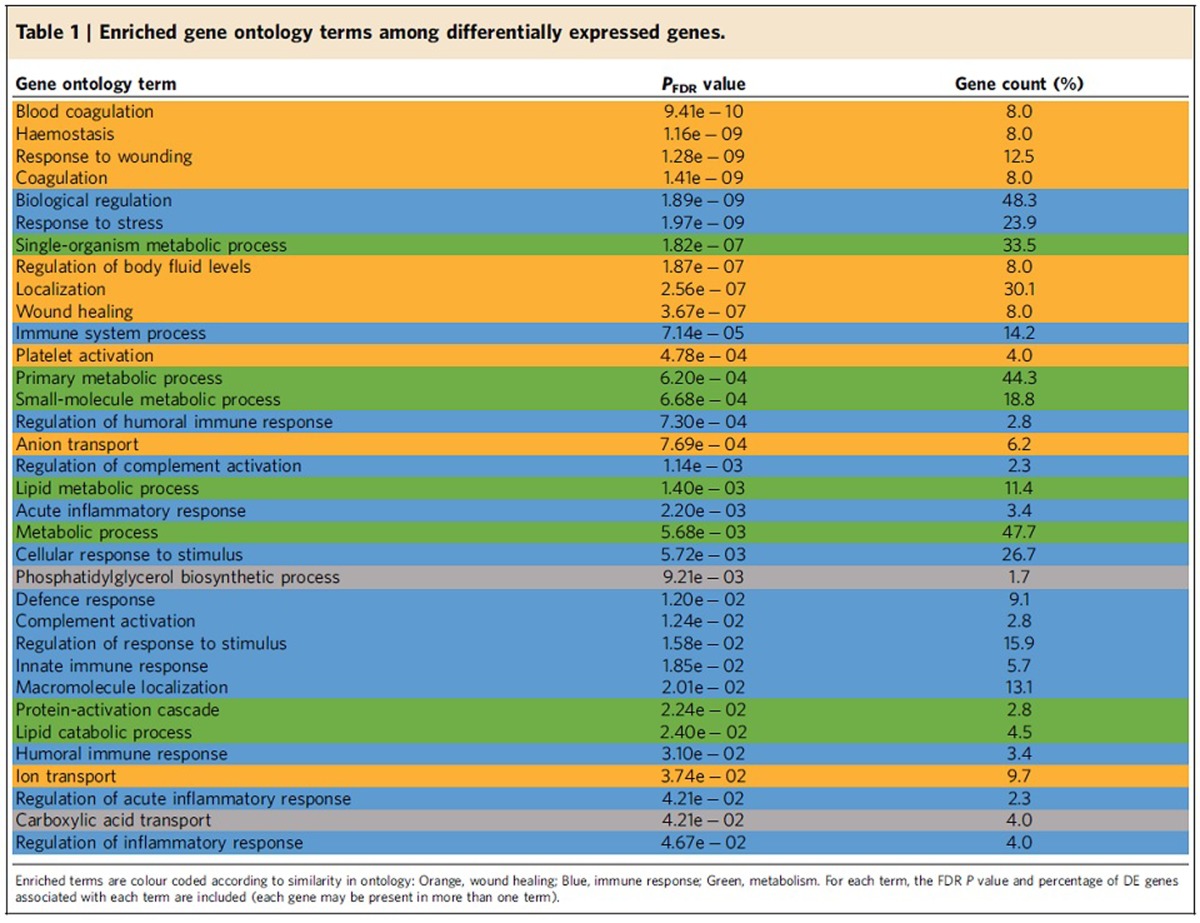
Enriched gene ontology terms among differentially expressed genes.
