# Supplementary material for: A single generation of domestication heritably alters the expression of hundreds of genes
Source: Nat Commun. 2016 Feb 17;7:10676. doi: 10.1038/ncomms10676 (PMC4757788; doi:10.1038/ncomms10676)
Supplement: Supplementary Information — Supplementary Figures 1-4 and Supplementary Table 1 [file ncomms10676-s1.pdf]

Supplementary Figures

Supplementary Figure 1: Illustration of the fish used and the types of crosses performed in this study. Both wild and first-generation (F1) hatchery fish that returned to spawn in the Hood River were collected for crosses in spring of 2010. Winter-run steelhead in the Hood River begin to return in December ("Run-year"), however, most return in the following calendar year and that is when spawning also occurs ("Brood-year"). Wild fish were born in the Hood River. Broodstock used to create hatchery fish were wild fish that were brought into the hatchery and spawned. Their offspring (F1 hatchery fish) were reared in a hatchery until approximately 1 year of age, at which point they were released near the adult spawning grounds. Both wild and first-generation hatchery fish then when out to sea for approximately 3 years and were subsequently collected at the Powerdale dam in run-year 2009 for the crosses used in this study. Numbers in the boxes represent the total numbers of individuals (and families in parentheses) used for RNA-Seq.

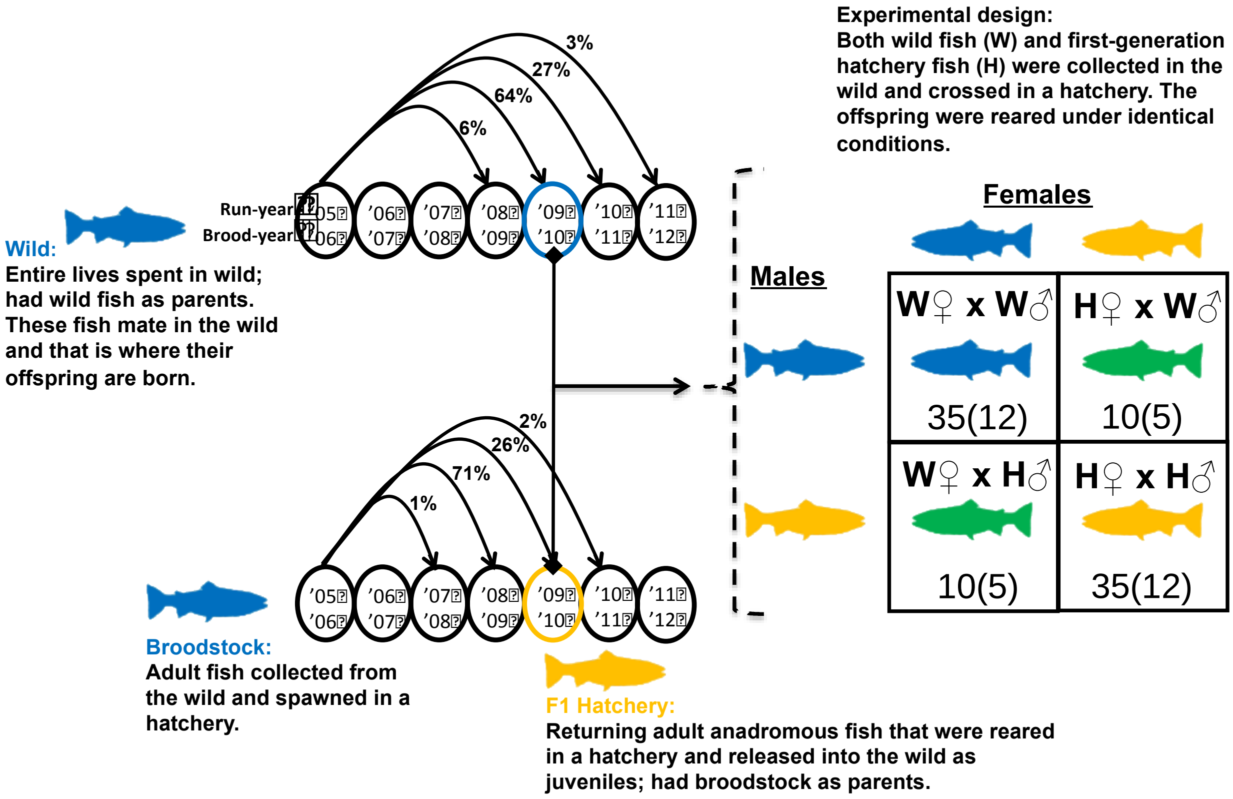

Supplementary Figure 2: Number of differentially expressed genes between the offspring for two types of comparisons where individuals were randomly sampled from a larger pool. In the first scenario, one offspring from each family of HxH and WxW parents was randomly sampled (see Table S1 for sample sizes). The total number of DE genes was calculated (FDR p-value < 0.05) and the process was repeated 100 times. The mean and 95% confidence intervals are shown (blue bar). We also identified the genetic sex of the offspring and examined the number of DE genes between males and females. Because there were more female than male offspring (Table S1), we randomly sampled the females so that the sample sizes were equivalent. This process was also repeated 100 times. There were large differences in gene expression between HxH and WxW offspring, but not between the sexes.

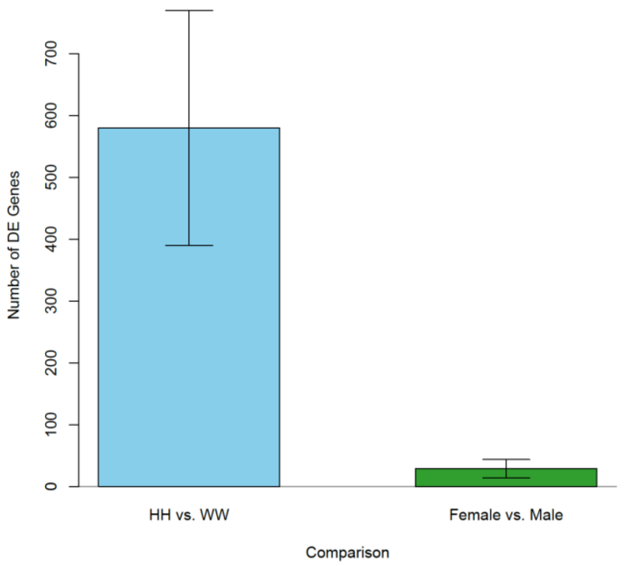

Supplementary Figure 3: Although the vast majority of DE genes did not show signatures of maternal effects (see Fig. 2), we were able to identify a handful of genes that show expression patterns consistent with maternal effects. These genes were identified *a posteriori* and include: Anoctamin-5, Apolipoprotein B-100, Beta-ureidopropionase, Bile salt export pump, Complement C1r subcomponent, Complement C3b alpha' chain, Galectin-9B, Methionine synthase, NADH-ubiquinone oxidoreductase chain 3, Rho-related GTP-binding protein RhoB, Solute carrier family 25 member 40, Stonustoxin subunit beta, V-type proton ATPase subunit d, 1VIP36-like protein, Zymogen granule membrane protein 16 (See Table S2).

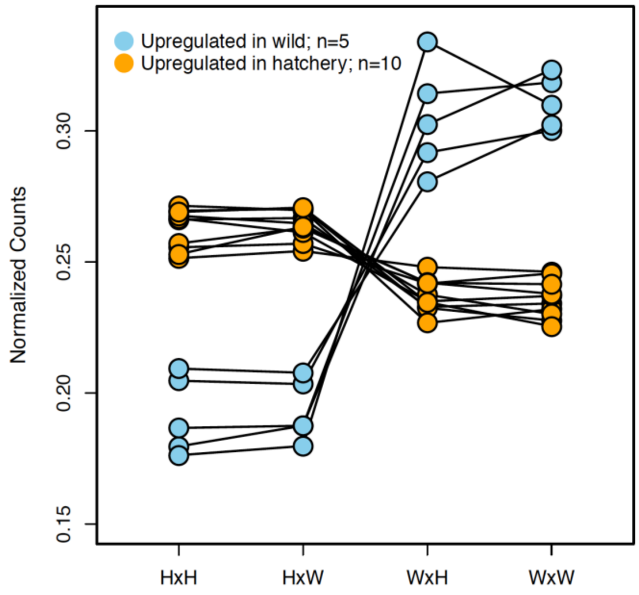

Supplementary Figure 4: Size of chromosome (in base pairs) versus the number of differentially expressed genes. Notice that there is a positive relationship between the size of the chromosome and the number of differentially expressed genes. Furthermore, DE genes were found on every chromosome. Because it is unlikely that all of these genes responded to selection across the genome, it is likely that selection acted on regulatory elements that affected downstream expression or via heritable epigenetic effects. Linear regression was highly significant ($p < 0.001$) with $R^2 = 0.377$. Dashed lines represent 95% confidence intervals. Chromosomes with more DE genes than expected given their size include chromosomes 2, 9, and 24, while those with fewer DE genes than expected include chromosomes 6, 11, 15, and 23.

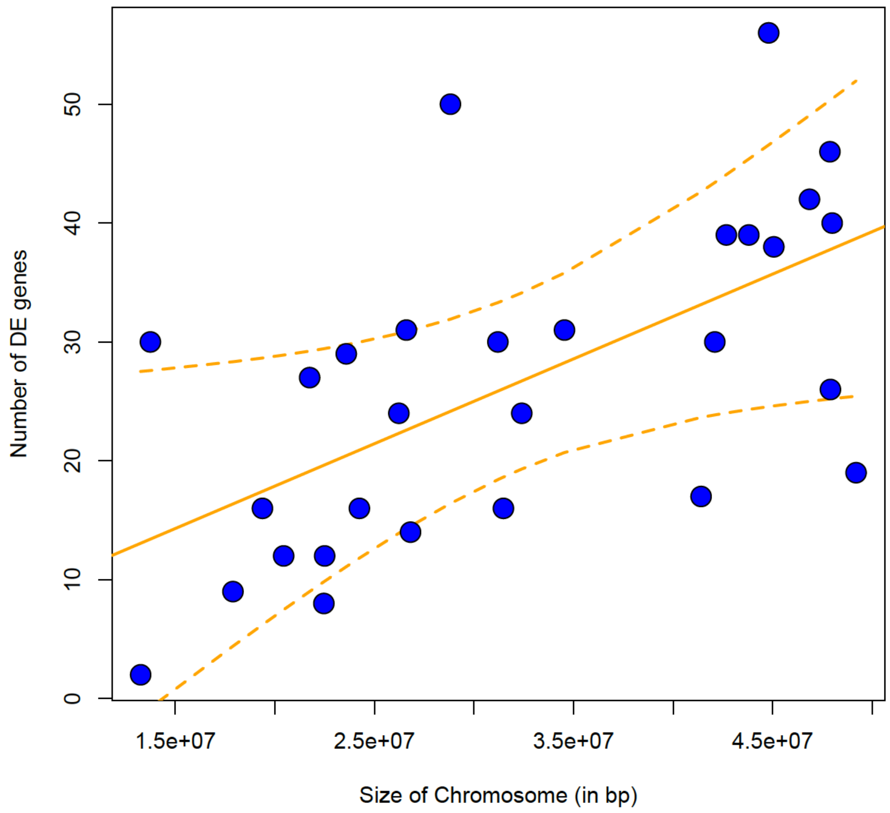

Supplementary Tables

Supplementary Table 1: A total of 34 crosses between two first-generation hatchery fish (HxH), two wild fish (WxW), and one hatchery and one wild fish (HxW and WxH) were performed at the hatchery. Six crosses were initially made between two wild fish (WxW) and two hatchery fish (HxH) (Panel A), from which we sequenced 4 offspring per cross type (2 males and 2 females; very few genes were DE between males and females – see Figure S2). To test for maternal effects, we also created matrices with all 4 possible cross types (Panel B). Here we created 5 matrices with all four cross types (WxW, WxH, HxW, HxH) and sequenced 2 offspring per cross (both female). We included one additional WxW and HxH offspring from an extra matrix to complete the flow cell.

Table S1: A

		Male	
		W	H
Female	W	WxW	
	H		HxH

Cross Type	Matrix	Crosses	Individuals	Sex(F/M)
WxW	1 to 6	6	24	12/12
HxH	1 to 6	6	24	12/12

Table S1: B

		Male	
		W	H
Female	W	WxW	WxH
	H	HxW	HxH

Cross Type	Matrix	Crosses	Individuals	Sex(F/M)
WxW	7 to 12	6	11	11/0
WxH	8 to 12	5	10	10/0
HxW	8 to 12	5	10	10/0
HxH	7 to 12	6	11	11/0
Totals:	12	34	90	66/24
