# Supplementary material for: A single generation of domestication heritably alters the expression of hundreds of genes
Source: Nat Commun. 2016 Feb 17;7:10676. doi: 10.1038/ncomms10676 (PMC4757788; doi:10.1038/ncomms10676)
Supplement: Supplementary Data 1 — Differentially expressed genes that were identified between the offspring of wild born fish (WxW) and the offspring of first-generation hatchery fish (HxH). Genes are sorted by log fold change (log FC). Also reported are the standardized protein names, full gene names, and false discovery rate adjusted p-value (FDR) for tests of differential expression. [file ncomms10676-s2.docx]

**Supplementary Data 1:** Differentially expressed genes that were identified between the offspring of wild born fish (WxW) and the offspring of first-generation hatchery fish (HxH). Genes are sorted by log fold change (log FC). Also reported are the standardized protein names, full gene names, and false discovery rate adjusted p-value (FDR) for tests of differential expression.

| **Num** | **Protein** | **DE gene** | **logFC** | **FDR** |
| --- | --- | --- | --- | --- |
| 1 | I7KJK9 | Trout C-polysaccharide binding protein 1, isoform 1 | -6.312E+00 | 5.800E-05 |
| 2 | C1QT3 | Complement C1q tumor necrosis factor-related protein 3 | -5.861E+00 | 1.955E-04 |
| 3 | CASPE | Caspase-14 | -5.060E+00 | 1.998E-02 |
| 4 | E3NQ84 | Putative uncharacterized protein | -4.963E+00 | 2.710E-04 |
| 5 | E3NQ84 | Putative uncharacterized protein | -4.187E+00 | 5.983E-03 |
| 6 | CASPE | Caspase-14 | -3.851E+00 | 2.203E-02 |
| 7 | M4A454 | Uncharacterized protein | -3.782E+00 | 9.526E-04 |
| 8 | HS12A | Heat shock 70 kDa protein 12A | -3.644E+00 | 2.531E-04 |
| 9 | M4A454 | Uncharacterized protein | -3.441E+00 | 4.287E-04 |
| 10 | E3NQ84 | Putative uncharacterized protein | -3.364E+00 | 4.293E-02 |
| 11 | M4A454 | Uncharacterized protein | -3.094E+00 | 9.110E-06 |
| 12 | M4A454 | Uncharacterized protein | -2.903E+00 | 9.450E-06 |
| 13 | RTXE | Probable RNA-directed DNA polymerase from transposon X-element | -2.785E+00 | 5.800E-05 |
| 14 | E9QED5 | Uncharacterized protein | -2.710E+00 | 7.887E-03 |
| 15 | RTXE | Probable RNA-directed DNA polymerase from transposon X-element | -2.624E+00 | 9.960E-05 |
| 16 | E9QED5 | Uncharacterized protein | -2.480E+00 | 8.145E-03 |
| 17 | RTXE | Probable RNA-directed DNA polymerase from transposon X-element | -2.474E+00 | 2.584E-04 |
| 18 | E9QED5 | Uncharacterized protein | -2.221E+00 | 6.411E-02 |
| 19 | E9QED5 | Uncharacterized protein | -2.180E+00 | 3.689E-02 |
| 20 | E9QED5 | Uncharacterized protein | -2.070E+00 | 6.520E-02 |
| 21 | E9QED5 | Uncharacterized protein | -2.015E+00 | 4.428E-02 |
| 22 | B9EQH7 | Zymogen granule membrane protein 16 | -1.482E+00 | 5.573E-03 |
| 23 | E9QED5 | Uncharacterized protein | -1.399E+00 | 5.260E-02 |
| 24 | M4AEG4 | Uncharacterized protein | -1.180E+00 | 1.066E-02 |
| 25 | NLRC3 | Protein NLRC3 | -1.102E+00 | 2.642E-04 |
| 26 | BRPF1 | Peregrin | -1.064E+00 | 5.116E-03 |
| 27 | RN115 | E3 ubiquitin-protein ligase RNF115 | -1.024E+00 | 1.786E-02 |
| 28 | POTE1 | Protection of telomeres protein 1 | -1.009E+00 | 5.425E-03 |
| 29 | TCB1 | Transposable element Tcb1 transposase | -9.825E-01 | 7.210E-06 |
| 30 | TIM50 | Mitochondrial import inner membrane translocase subunit TIM50 | -9.813E-01 | 1.415E-02 |
| 31 | CYB | Cytochrome b | -9.621E-01 | 7.107E-02 |
| 32 | NU2M | NADH-ubiquinone oxidoreductase chain 2 | -9.491E-01 | 9.649E-03 |
| 33 | NU5M | NADH-ubiquinone oxidoreductase chain 5 | -9.340E-01 | 4.297E-02 |
| 34 | COA4 | Cytochrome c oxidase assembly factor 4 homolog, mitochondrial | -9.283E-01 | 4.825E-03 |
| 35 | NU3M | NADH-ubiquinone oxidoreductase chain 3 | -9.193E-01 | 5.116E-03 |
| 36 | MTG1 | Mitochondrial ribosome-associated GTPase 1 | -9.189E-01 | 1.250E-02 |
| 37 | NUP53 | Nucleoporin NUP53 | -8.844E-01 | 2.227E-02 |
| 38 | R7UUG5 | Uncharacterized protein | -8.658E-01 | 9.780E-03 |
| 39 | NU5M | NADH-ubiquinone oxidoreductase chain 5 | -8.632E-01 | 1.878E-02 |
| 40 | DHI1L | Hydroxysteroid 11-beta-dehydrogenase 1-like protein | -8.489E-01 | 1.437E-03 |
| 41 | BUP1 | Beta-ureidopropionase | -8.447E-01 | 5.800E-05 |
| 42 | CLC4E | C-type lectin domain family 4 member E | -8.262E-01 | 3.701E-02 |
| 43 | STN1 | CST complex subunit STN1 | -7.997E-01 | 9.056E-03 |
| 44 | H3IDA3 | Uncharacterized protein | -7.895E-01 | 4.792E-02 |
| 45 | NU5M | NADH-ubiquinone oxidoreductase chain 5 | -7.878E-01 | 6.666E-02 |
| 46 | M4AZS3 | Uncharacterized protein | -7.717E-01 | 5.668E-03 |
| 47 | HHLA2 | HERV-H LTR-associating protein 2 | -7.661E-01 | 9.500E-05 |
| 48 | TRIQK | Triple QxxK/R motif-containing protein | -7.652E-01 | 2.462E-03 |
| 49 | ITB1 | Integrin beta-1 | -7.472E-01 | 1.420E-02 |
| 50 | YB039 | Uncharacterized protein LINC00116 homolog | -7.308E-01 | 1.303E-04 |
| 51 | FKB14 | Peptidyl-prolyl cis-trans isomerase FKBP14 | -7.144E-01 | 3.946E-02 |
| 52 | DRXIA | Draxin-A | -7.133E-01 | 2.870E-02 |
| 53 | C0HA44 | CD99 antigen | -7.097E-01 | 5.221E-02 |
| 54 | IN80E | INO80 complex subunit E | -7.090E-01 | 4.779E-02 |
| 55 | JHD2C | Probable JmjC domain-containing histone demethylation protein 2C | -7.076E-01 | 7.210E-06 |
| 56 | OAZ1 | Ornithine decarboxylase antizyme 1 | -6.780E-01 | 5.130E-02 |
| 57 | EAF1 | ELL-associated factor 1 | -6.747E-01 | 5.249E-02 |
| 58 | YB039 | Uncharacterized protein LINC00116 homolog | -6.653E-01 | 2.323E-02 |
| 59 | PI42C | Phosphatidylinositol 5-phosphate 4-kinase type-2 gamma | -6.634E-01 | 5.789E-02 |
| 60 | TAF1A | TATA box-binding protein-associated factor RNA polymerase I subunit A | -6.593E-01 | 2.819E-02 |
| 61 | LEG9B | Galectin-9B | -6.578E-01 | 9.322E-03 |
| 62 | DHI1L | Hydroxysteroid 11-beta-dehydrogenase 1-like protein | -6.568E-01 | 3.048E-02 |
| 63 | B2DBF2 | Troponin I | -6.565E-01 | 1.910E-02 |
| 64 | F6KMM9 | NACHT, LRR and PYD domains-containing protein | -6.439E-01 | 3.484E-02 |
| 65 | HHLA2 | HERV-H LTR-associating protein 2 | -6.431E-01 | 2.597E-02 |
| 66 | NU1M | NADH-ubiquinone oxidoreductase chain 1 | -6.351E-01 | 6.780E-02 |
| 67 | TIM13 | Mitochondrial import inner membrane translocase subunit Tim13 | -6.342E-01 | 3.792E-02 |
| 68 | PDGFC | Platelet-derived growth factor C, receptor-binding form | -6.339E-01 | 6.303E-02 |
| 69 | HHLA2 | HERV-H LTR-associating protein 2 | -6.272E-01 | 5.934E-02 |
| 70 | YB039 | Uncharacterized protein LINC00116 homolog | -6.224E-01 | 5.030E-03 |
| 71 | DEK | Protein DEK | -6.180E-01 | 2.717E-02 |
| 72 | STN1 | CST complex subunit STN1 | -6.131E-01 | 5.688E-02 |
| 73 | SH3L3 | SH3 domain-binding glutamic acid-rich-like protein 3 | -6.087E-01 | 6.461E-03 |
| 74 | PIM1 | Serine/threonine-protein kinase pim-1 | -6.032E-01 | 2.584E-04 |
| 75 | MSPE | Beta-microseminoprotein E1 | -5.996E-01 | 1.726E-02 |
| 76 | PG12B | Group XIIB secretory phospholipase A2-like protein | -5.883E-01 | 2.969E-02 |
| 77 | PG12B | Group XIIB secretory phospholipase A2-like protein | -5.866E-01 | 1.402E-02 |
| 78 | CYTB | Cystatin-B | -5.843E-01 | 1.734E-02 |
| 79 | CYTB | Cystatin-B | -5.817E-01 | 6.134E-02 |
| 80 | CYH1 | Cytohesin-1 | -5.715E-01 | 6.461E-03 |
| 81 | NUA4L | NADH dehydrogenase [ubiquinone] 1 alpha subcomplex subunit 4-like 2 | -5.629E-01 | 6.097E-02 |
| 82 | GILT | Gamma-interferon-inducible lysosomal thiol reductase | -5.602E-01 | 5.805E-02 |
| 83 | NOE3 | Noelin-3 | -5.601E-01 | 6.461E-03 |
| 84 | RPAP2 | Putative RNA polymerase II subunit B1 CTD phosphatase rpap2 | -5.466E-01 | 3.317E-02 |
| 85 | HAOX2 | Hydroxyacid oxidase 2 | -5.455E-01 | 7.995E-03 |
| 86 | WDR73 | WD repeat-containing protein 73 | -5.453E-01 | 3.102E-02 |
| 87 | Q5DHF4 | SJCHGC04881 protein | -5.441E-01 | 3.875E-02 |
| 88 | STK38 | Serine/threonine-protein kinase 38 | -5.440E-01 | 2.557E-02 |
| 89 | GULP1 | PTB domain-containing engulfment adapter protein 1 | -5.411E-01 | 2.968E-02 |
| 90 | CYTB | Cystatin-B | -5.356E-01 | 4.817E-02 |
| 91 | S2611 | Sodium-independent sulfate anion transporter | -5.316E-01 | 2.025E-02 |
| 92 | RB27B | Ras-related protein Rab-27B | -5.313E-01 | 5.117E-03 |
| 93 | S18L2 | SS18-like protein 2 | -5.270E-01 | 2.731E-02 |
| 94 | KAT8 | Histone acetyltransferase KAT8 | -5.222E-01 | 5.242E-04 |
| 95 | COX8B | Cytochrome c oxidase subunit 8B, mitochondrial | -5.126E-01 | 1.492E-02 |
| 96 | GULP1 | PTB domain-containing engulfment adapter protein 1 | -5.106E-01 | 4.817E-02 |
| 97 | KIFA3 | Kinesin-associated protein 3 | -5.083E-01 | 1.908E-02 |
| 98 | TRIL | TLR4 interactor with leucine rich repeats | -5.062E-01 | 6.219E-02 |
| 99 | CYTB | Cystatin-B | -5.051E-01 | 6.219E-02 |
| 100 | TNNI1 | Troponin I, slow skeletal muscle | -5.030E-01 | 6.666E-03 |
| 101 | NAT9 | N-acetyltransferase 9 | -5.028E-01 | 4.186E-03 |
| 102 | C6EWZ5 | FGF2 | -5.027E-01 | 6.780E-02 |
| 103 | GBP1 | Interferon-induced guanylate-binding protein 1 | -5.015E-01 | 2.717E-02 |
| 104 | OTOR | Otoraplin | -5.004E-01 | 1.336E-02 |
| 105 | IKZF5 | Zinc finger protein Pegasus | -4.983E-01 | 4.278E-02 |
| 106 | LIN52 | Protein lin-52 homolog | -4.936E-01 | 2.089E-02 |
| 107 | TT39B | Tetratricopeptide repeat protein 39B | -4.929E-01 | 3.689E-02 |
| 108 | PDIA2 | Protein disulfide-isomerase A2 | -4.896E-01 | 2.719E-02 |
| 109 | PSMD4 | 26S proteasome non-ATPase regulatory subunit 4 | -4.885E-01 | 1.524E-02 |
| 110 | DFP | Putative defense protein Hdd11-like | -4.868E-01 | 1.713E-02 |
| 111 | OPA1 | Dynamin-like 120 kDa protein, form S1 | -4.866E-01 | 3.233E-02 |
| 112 | GHC1 | Mitochondrial glutamate carrier 1 | -4.833E-01 | 1.388E-02 |
| 113 | UNG | Uracil-DNA glycosylase | -4.828E-01 | 6.665E-02 |
| 114 | ANX13 | Annexin A13 | -4.762E-01 | 4.940E-03 |
| 115 | XPO2 | Exportin-2 | -4.736E-01 | 3.484E-02 |
| 116 | OTOR | Otoraplin | -4.722E-01 | 9.041E-03 |
| 117 | TOR4A | Torsin-4A | -4.697E-01 | 3.717E-02 |
| 118 | SMIM8 | Small integral membrane protein 8 | -4.676E-01 | 3.484E-02 |
| 119 | NSMA3 | Sphingomyelin phosphodiesterase 4 | -4.670E-01 | 6.769E-02 |
| 120 | RB27B | Ras-related protein Rab-27B | -4.669E-01 | 2.801E-02 |
| 121 | DHX37 | Probable ATP-dependent RNA helicase DHX37 | -4.654E-01 | 3.144E-02 |
| 122 | BEND6 | BEN domain-containing protein 6 | -4.648E-01 | 2.664E-02 |
| 123 | RCC1 | Regulator of chromosome condensation | -4.639E-01 | 5.763E-02 |
| 124 | SESD1 | SEC14 domain and spectrin repeat-containing protein 1 | -4.621E-01 | 4.312E-02 |
| 125 | OTOR | Otoraplin | -4.613E-01 | 1.485E-02 |
| 126 | CREL2 | Cysteine-rich with EGF-like domain protein 2 | -4.606E-01 | 3.954E-02 |
| 127 | CN166 | UPF0568 protein C14orf166 homolog | -4.589E-01 | 4.864E-02 |
| 128 | IKZF5 | Zinc finger protein Pegasus | -4.567E-01 | 7.024E-02 |
| 129 | STX4 | Syntaxin-4 | -4.550E-01 | 2.397E-02 |
| 130 | TNNT2 | Troponin T, cardiac muscle isoforms | -4.511E-01 | 4.211E-02 |
| 131 | B5X3H6 | LYRIC | -4.502E-01 | 6.154E-02 |
| 132 | RHOB | Rho-related GTP-binding protein RhoB | -4.474E-01 | 1.485E-02 |
| 133 | QCR6 | Cytochrome b-c1 complex subunit 6, mitochondrial | -4.424E-01 | 7.090E-02 |
| 134 | CHPT1 | Cholinephosphotransferase 1 | -4.407E-01 | 4.817E-02 |
| 135 | TNNI1 | Troponin I, slow skeletal muscle | -4.403E-01 | 3.374E-02 |
| 136 | Q5C6B2 | SJCHGC04011 protein | -4.402E-01 | 6.723E-02 |
| 137 | SH3L3 | SH3 domain-binding glutamic acid-rich-like protein 3 | -4.387E-01 | 5.174E-04 |
| 138 | PIM1 | Serine/threonine-protein kinase pim-1 | -4.381E-01 | 2.696E-03 |
| 139 | DPOD4 | DNA polymerase delta subunit 4 | -4.375E-01 | 2.364E-02 |
| 140 | PIM1 | Serine/threonine-protein kinase pim-1 | -4.370E-01 | 6.576E-02 |
| 141 | H3CL73 | Uncharacterized protein | -4.345E-01 | 3.347E-02 |
| 142 | PTGR1 | Prostaglandin reductase 1 | -4.325E-01 | 2.992E-02 |
| 143 | BAP29 | B-cell receptor-associated protein 29 | -4.309E-01 | 7.995E-03 |
| 144 | ZN271 | Zinc finger protein 271 | -4.294E-01 | 2.479E-02 |
| 145 | GLOD4 | Glyoxalase domain-containing protein 4 | -4.284E-01 | 2.450E-02 |
| 146 | TACC3 | Transforming acidic coiled-coil-containing protein 3 | -4.260E-01 | 4.012E-02 |
| 147 | SUMO3 | Small ubiquitin-related modifier 3 | -4.237E-01 | 4.297E-02 |
| 148 | MYC | Transcriptional regulator Myc | -4.220E-01 | 5.083E-02 |
| 149 | ATOH8 | Protein atonal homolog 8 | -4.200E-01 | 3.789E-02 |
| 150 | JADE3 | Protein Jade-3 | -4.166E-01 | 2.835E-02 |
| 151 | IFRD1 | Interferon-related developmental regulator 1 | -4.166E-01 | 7.229E-03 |
| 152 | DUS23 | Dual specificity protein phosphatase 23 | -4.151E-01 | 4.979E-02 |
| 153 | EMID1 | EMI domain-containing protein 1 | -4.130E-01 | 5.860E-02 |
| 154 | SRSF5 | Serine/arginine-rich splicing factor 5 | -4.129E-01 | 2.917E-02 |
| 155 | IER5L | Immediate early response gene 5-like protein | -4.126E-01 | 6.868E-02 |
| 156 | A0P9K9 | Tc1-like transporase | -4.111E-01 | 1.041E-02 |
| 157 | TGDS | dTDP-D-glucose 4,6-dehydratase | -4.109E-01 | 2.230E-02 |
| 158 | PIR | Pirin | -4.095E-01 | 5.976E-02 |
| 159 | B2MG | Beta-2-microglobulin | -4.094E-01 | 2.120E-02 |
| 160 | DC2L1 | Cytoplasmic dynein 2 light intermediate chain 1 | -4.087E-01 | 2.479E-02 |
| 161 | PIM1 | Serine/threonine-protein kinase pim-1 | -4.059E-01 | 2.178E-02 |
| 162 | P2RX5 | P2X purinoceptor 5 | -4.056E-01 | 2.423E-02 |
| 163 | TNNI1 | Troponin I, slow skeletal muscle | -4.041E-01 | 2.219E-02 |
| 164 | COPZ1 | Coatomer subunit zeta-1 | -4.021E-01 | 3.447E-02 |
| 165 | COA3 | Cytochrome c oxidase assembly protein 3 homolog, mitochondrial | -3.997E-01 | 5.691E-02 |
| 166 | B2MG | Beta-2-microglobulin | -3.984E-01 | 1.726E-02 |
| 167 | DAZP1 | DAZ-associated protein 1 | -3.973E-01 | 2.292E-02 |
| 168 | TPPC2 | Trafficking protein particle complex subunit 2 | -3.963E-01 | 5.748E-02 |
| 169 | E7F3G4 | Uncharacterized protein | -3.941E-01 | 6.036E-02 |
| 170 | ATP5J | ATP synthase-coupling factor 6, mitochondrial | -3.940E-01 | 2.969E-02 |
| 171 | EMC9 | ER membrane protein complex subunit 9 | -3.934E-01 | 2.203E-02 |
| 172 | TMED5 | Transmembrane emp24 domain-containing protein 5 | -3.931E-01 | 2.490E-02 |
| 173 | KCC2D | Calcium/calmodulin-dependent protein kinase type II subunit delta | -3.922E-01 | 5.531E-02 |
| 174 | M4A15 | Membrane-spanning 4-domains subfamily A member 15 | -3.902E-01 | 2.030E-02 |
| 175 | CUTA | Protein CutA homolog | -3.897E-01 | 5.249E-02 |
| 176 | ING4 | Inhibitor of growth protein 4 | -3.885E-01 | 2.480E-02 |
| 177 | ITA11 | Integrin alpha-11 | -3.857E-01 | 5.851E-02 |
| 178 | ETS1 | Protein C-ets-1 | -3.835E-01 | 1.415E-02 |
| 179 | MAGG1 | Melanoma-associated antigen G1 | -3.801E-01 | 5.130E-02 |
| 180 | ZN567 | Zinc finger protein 567 | -3.789E-01 | 6.040E-02 |
| 181 | CATZ | Cathepsin Z | -3.786E-01 | 5.688E-02 |
| 182 | RTN3 | Reticulon-3 | -3.774E-01 | 9.501E-03 |
| 183 | MAFB | Transcription factor MafB | -3.719E-01 | 2.147E-02 |
| 184 | B2MG | Beta-2-microglobulin | -3.705E-01 | 2.854E-02 |
| 185 | H2MK54 | Uncharacterized protein | -3.653E-01 | 3.551E-02 |
| 186 | SMI19 | Small integral membrane protein 19 | -3.638E-01 | 3.598E-02 |
| 187 | C6EWZ5 | FGF2 | -3.636E-01 | 5.591E-02 |
| 188 | TOM5 | Mitochondrial import receptor subunit TOM5 homolog | -3.598E-01 | 4.278E-02 |
| 189 | DESI1 | Desumoylating isopeptidase 1 | -3.594E-01 | 2.115E-02 |
| 190 | NFIP1 | NEDD4 family-interacting protein 1 | -3.525E-01 | 2.523E-02 |
| 191 | HSBP1 | Heat shock factor-binding protein 1 | -3.494E-01 | 5.387E-02 |
| 192 | CJ032 | UPF0693 protein C10orf32 homolog | -3.490E-01 | 4.257E-02 |
| 193 | MYADM | Myeloid-associated differentiation marker homolog | -3.433E-01 | 3.524E-02 |
| 194 | B2MG | Beta-2-microglobulin | -3.426E-01 | 3.551E-02 |
| 195 | LIN1 | LINE-1 reverse transcriptase homolog | -3.418E-01 | 4.864E-02 |
| 196 | BAP29 | B-cell receptor-associated protein 29 | -3.418E-01 | 5.593E-02 |
| 197 | B2MG | Beta-2-microglobulin | -3.400E-01 | 6.606E-02 |
| 198 | BAP29 | B-cell receptor-associated protein 29 | -3.398E-01 | 6.877E-02 |
| 199 | IFRD1 | Interferon-related developmental regulator 1 | -3.382E-01 | 2.578E-02 |
| 200 | RALB | Ras-related protein Ral-B | -3.328E-01 | 1.524E-02 |
| 201 | RNKA | Ribonuclease kappa-A | -3.300E-01 | 8.287E-03 |
| 202 | RNKA | Ribonuclease kappa-A | -3.287E-01 | 2.292E-02 |
| 203 | COX8B | Cytochrome c oxidase subunit 8B, mitochondrial | -3.265E-01 | 5.851E-02 |
| 204 | IFT80 | Intraflagellar transport protein 80 homolog | -3.251E-01 | 5.688E-02 |
| 205 | CRIP1 | Cysteine-rich protein 1 | -3.230E-01 | 6.097E-02 |
| 206 | EAF6 | Chromatin modification-related protein MEAF6 | -3.181E-01 | 7.152E-02 |
| 207 | MARCS | Myristoylated alanine-rich C-kinase substrate | -3.120E-01 | 4.864E-02 |
| 208 | CQ067 | Uncharacterized protein C17orf67 homolog | -3.063E-01 | 7.140E-02 |
| 209 | PIM1 | Serine/threonine-protein kinase pim-1 | -3.037E-01 | 4.768E-02 |
| 210 | CLIC4 | Chloride intracellular channel protein 4 | -3.018E-01 | 3.777E-02 |
| 211 | QCR8 | Cytochrome b-c1 complex subunit 8 | -3.005E-01 | 6.773E-02 |
| 212 | SELT2 | Selenoprotein T2 | -2.978E-01 | 2.030E-02 |
| 213 | PMP22 | Peripheral myelin protein 22 | -2.976E-01 | 5.319E-02 |
| 214 | CHMP3 | Charged multivesicular body protein 3 | -2.965E-01 | 4.567E-02 |
| 215 | AN32A | Acidic leucine-rich nuclear phosphoprotein 32 family member A | -2.962E-01 | 5.533E-02 |
| 216 | ACN9 | Protein ACN9 homolog, mitochondrial | -2.947E-01 | 6.768E-02 |
| 217 | DSS1 | 26S proteasome complex subunit DSS1 | -2.938E-01 | 5.691E-02 |
| 218 | SYN1L | Synapse differentiation-inducing gene protein 1-like | -2.914E-01 | 2.998E-02 |
| 219 | RAC2 | Ras-related C3 botulinum toxin substrate 2 | -2.827E-01 | 6.528E-02 |
| 220 | SRSF2 | Serine/arginine-rich splicing factor 2 | -2.812E-01 | 3.068E-02 |
| 221 | ATPF1 | ATP synthase mitochondrial F1 complex assembly factor 1 | -2.788E-01 | 6.097E-02 |
| 222 | RALB | Ras-related protein Ral-B | -2.742E-01 | 2.446E-02 |
| 223 | AP1M1 | AP-1 complex subunit mu-1 | -2.655E-01 | 2.748E-02 |
| 224 | TX1B3 | Tax1-binding protein 3 | -2.648E-01 | 6.154E-02 |
| 225 | TGT | Queuine tRNA-ribosyltransferase | -2.615E-01 | 6.229E-02 |
| 226 | ARPC4 | Actin-related protein 2/3 complex subunit 4 | -2.544E-01 | 6.175E-02 |
| 227 | Q4SRN3 | Uncharacterized protein | -2.522E-01 | 4.813E-02 |
| 228 | COTL1 | Coactosin-like protein | -2.464E-01 | 2.275E-02 |
| 229 | SYN1L | Synapse differentiation-inducing gene protein 1-like | -2.422E-01 | 7.162E-02 |
| 230 | ZN148 | Zinc finger protein 148 | -2.393E-01 | 5.922E-02 |
| 231 | FSTL1 | Follistatin-related protein 1 | -2.284E-01 | 4.704E-02 |
| 232 | SYN1L | Synapse differentiation-inducing gene protein 1-like | -1.615E-01 | 7.162E-02 |
| 233 | DHE3 | Glutamate dehydrogenase, mitochondrial | 2.310E-01 | 5.743E-02 |
| 234 | DHE3 | Glutamate dehydrogenase, mitochondrial | 2.340E-01 | 6.693E-02 |
| 235 | ACON | Aconitate hydratase, mitochondrial | 2.484E-01 | 6.780E-02 |
| 236 | DHE3 | Glutamate dehydrogenase, mitochondrial | 2.568E-01 | 5.260E-02 |
| 237 | DCAM | S-adenosylmethionine decarboxylase alpha chain | 2.586E-01 | 6.693E-02 |
| 238 | EF1A0 | Elongation factor 1-alpha, somatic form | 2.625E-01 | 5.130E-02 |
| 239 | ADK | Adenosine kinase | 2.700E-01 | 6.724E-02 |
| 240 | DHSA | Succinate dehydrogenase [ubiquinone] flavoprotein subunit, mitochondrial | 2.720E-01 | 5.572E-02 |
| 241 | MADD | MAP kinase-activating death domain protein | 2.736E-01 | 3.701E-02 |
| 242 | AMBP | Alpha-1-microglobulin | 2.857E-01 | 4.116E-02 |
| 243 | TMM19 | Transmembrane protein 19 | 2.904E-01 | 4.881E-02 |
| 244 | AMBP | Alpha-1-microglobulin | 3.004E-01 | 6.780E-02 |
| 245 | ENPP2 | Ectonucleotide pyrophosphatase/phosphodiesterase family member 2 | 3.017E-01 | 6.356E-02 |
| 246 | SARDH | Sarcosine dehydrogenase, mitochondrial | 3.076E-01 | 8.102E-03 |
| 247 | KPYM | Pyruvate kinase PKM | 3.097E-01 | 3.310E-02 |
| 248 | OCTC | Peroxisomal carnitine O-octanoyltransferase | 3.101E-01 | 3.703E-02 |
| 249 | OCTC | Peroxisomal carnitine O-octanoyltransferase | 3.111E-01 | 4.603E-02 |
| 250 | RDHE2 | Epidermal retinol dehydrogenase 2 | 3.125E-01 | 2.717E-02 |
| 251 | MYH11 | Myosin-11 | 3.125E-01 | 4.817E-02 |
| 252 | DYN1 | Dynamin-1 | 3.160E-01 | 1.541E-02 |
| 253 | CO8G | Complement component C8 gamma chain | 3.177E-01 | 7.105E-02 |
| 254 | SQSTM | Sequestosome-1 | 3.198E-01 | 6.297E-02 |
| 255 | IAH1 | Isoamyl acetate-hydrolyzing esterase 1 homolog | 3.239E-01 | 4.817E-02 |
| 256 | VPP1 | V-type proton ATPase 116 kDa subunit a isoform 1 | 3.245E-01 | 6.264E-02 |
| 257 | DHE3 | Glutamate dehydrogenase, mitochondrial | 3.247E-01 | 4.996E-02 |
| 258 | IDHC | Isocitrate dehydrogenase [NADP] cytoplasmic | 3.261E-01 | 2.785E-02 |
| 259 | S27A1 | Long-chain fatty acid transport protein 1 | 3.271E-01 | 4.979E-02 |
| 260 | VWF | von Willebrand factor | 3.273E-01 | 3.689E-02 |
| 261 | MAP4 | Microtubule-associated protein 4 | 3.273E-01 | 3.655E-02 |
| 262 | MYH11 | Myosin-11 | 3.274E-01 | 5.481E-02 |
| 263 | KPYM | Pyruvate kinase PKM | 3.288E-01 | 1.649E-02 |
| 264 | AMBP | Alpha-1-microglobulin | 3.309E-01 | 4.312E-02 |
| 265 | S27A1 | Long-chain fatty acid transport protein 1 | 3.311E-01 | 5.889E-02 |
| 266 | MGLL | Monoglyceride lipase | 3.313E-01 | 2.717E-02 |
| 267 | HACL1 | 2-hydroxyacyl-CoA lyase 1 | 3.323E-01 | 6.804E-02 |
| 268 | OCTC | Peroxisomal carnitine O-octanoyltransferase | 3.330E-01 | 5.688E-02 |
| 269 | PJA2 | E3 ubiquitin-protein ligase Praja-2 | 3.337E-01 | 5.688E-02 |
| 270 | ANT3 | Antithrombin-III | 3.340E-01 | 4.020E-02 |
| 271 | VPP1 | V-type proton ATPase 116 kDa subunit a isoform 1 | 3.346E-01 | 4.211E-02 |
| 272 | CP27A | Sterol 26-hydroxylase, mitochondrial | 3.369E-01 | 5.691E-02 |
| 273 | 4F2 | 4F2 cell-surface antigen heavy chain | 3.371E-01 | 3.780E-02 |
| 274 | ISL3 | Insulin gene enhancer protein ISL-3 | 3.389E-01 | 6.768E-02 |
| 275 | NEC2 | Neuroendocrine convertase 2 | 3.445E-01 | 6.297E-02 |
| 276 | OTX5B | Homeobox protein otx5-B | 3.446E-01 | 3.767E-02 |
| 277 | SCLY | Selenocysteine lyase | 3.460E-01 | 3.438E-02 |
| 278 | PYGM | Glycogen phosphorylase, muscle form | 3.462E-01 | 5.533E-02 |
| 279 | APOB | Apolipoprotein B-48 | 3.462E-01 | 5.674E-02 |
| 280 | CK024 | Uncharacterized protein C11orf24 homolog | 3.468E-01 | 1.415E-02 |
| 281 | ENTP6 | Ectonucleoside triphosphate diphosphohydrolase 6 | 3.468E-01 | 4.817E-02 |
| 282 | DHE3 | Glutamate dehydrogenase, mitochondrial | 3.472E-01 | 4.020E-02 |
| 283 | AMBP | Alpha-1-microglobulin | 3.472E-01 | 2.737E-02 |
| 284 | CLUA1 | Clusterin-associated protein 1 homolog | 3.475E-01 | 6.546E-02 |
| 285 | MRS2 | Magnesium transporter MRS2 homolog, mitochondrial | 3.479E-01 | 2.969E-02 |
| 286 | PYGL | Glycogen phosphorylase, liver form | 3.480E-01 | 7.160E-03 |
| 287 | QSOX1 | Sulfhydryl oxidase 1 | 3.493E-01 | 2.968E-02 |
| 288 | ANT3 | Antithrombin-III | 3.499E-01 | 5.151E-02 |
| 289 | NPDC1 | Neural proliferation differentiation and control protein 1 | 3.500E-01 | 5.851E-02 |
| 290 | NCPR | NADPH--cytochrome P450 reductase | 3.510E-01 | 5.851E-02 |
| 291 | CPXM2 | Inactive carboxypeptidase-like protein X2 | 3.526E-01 | 3.145E-02 |
| 292 | M3K5 | Mitogen-activated protein kinase 5 | 3.539E-01 | 5.260E-02 |
| 293 | PJA2 | E3 ubiquitin-protein ligase Praja-2 | 3.542E-01 | 3.068E-02 |
| 294 | IDHC | Isocitrate dehydrogenase [NADP] cytoplasmic | 3.545E-01 | 1.229E-02 |
| 295 | VPP1 | V-type proton ATPase 116 kDa subunit a isoform 1 | 3.559E-01 | 2.331E-02 |
| 296 | GYS2 | Glycogen [starch] synthase, liver | 3.595E-01 | 5.833E-02 |
| 297 | TRFE | Serotransferrin | 3.599E-01 | 6.012E-02 |
| 298 | ZDH14 | Probable palmitoyltransferase ZDHHC14 | 3.604E-01 | 5.617E-02 |
| 299 | MA2B1 | Lysosomal alpha-mannosidase | 3.617E-01 | 3.790E-02 |
| 300 | AMBP | Alpha-1-microglobulin | 3.620E-01 | 2.026E-02 |
| 301 | ENTP6 | Ectonucleoside triphosphate diphosphohydrolase 6 | 3.626E-01 | 5.336E-02 |
| 302 | MA2B1 | Lysosomal alpha-mannosidase | 3.631E-01 | 4.268E-02 |
| 303 | A1AT | Alpha-1-antitrypsin | 3.634E-01 | 5.657E-02 |
| 304 | F172A | Protein FAM172A | 3.638E-01 | 6.521E-02 |
| 305 | PJA2 | E3 ubiquitin-protein ligase Praja-2 | 3.639E-01 | 3.920E-02 |
| 306 | DYST | Dystonin | 3.641E-01 | 4.347E-02 |
| 307 | FA7 | Factor VII light chain | 3.646E-01 | 5.383E-02 |
| 308 | DCE1 | Glutamate decarboxylase 1 | 3.655E-01 | 5.832E-02 |
| 309 | PLPL9 | 85/88 kDa calcium-independent phospholipase A2 | 3.660E-01 | 4.704E-02 |
| 310 | ETBR2 | Endothelin B receptor-like protein 2 | 3.661E-01 | 5.418E-02 |
| 311 | MARK2 | Serine/threonine-protein kinase MARK2 | 3.668E-01 | 6.498E-02 |
| 312 | PYGL | Glycogen phosphorylase, liver form | 3.681E-01 | 1.138E-02 |
| 313 | ADCK4 | Uncharacterized aarF domain-containing protein kinase 4 | 3.682E-01 | 5.918E-02 |
| 314 | LAT4 | Large neutral amino acids transporter small subunit 4 | 3.684E-01 | 7.030E-02 |
| 315 | INP5K | Inositol polyphosphate 5-phosphatase K | 3.694E-01 | 5.691E-02 |
| 316 | ALBU1 | Serum albumin 1 | 3.694E-01 | 4.187E-02 |
| 317 | GPX3 | Glutathione peroxidase 3 | 3.700E-01 | 2.230E-02 |
| 318 | CAP2 | Adenylyl cyclase-associated protein 2 | 3.713E-01 | 5.640E-02 |
| 319 | FA7 | Coagulation factor VII | 3.718E-01 | 2.968E-02 |
| 320 | ALBU2 | Serum albumin 2 | 3.723E-01 | 5.640E-02 |
| 321 | Q49BZ3 | Calpastatin long | 3.723E-01 | 2.230E-02 |
| 322 | PYGL | Glycogen phosphorylase, liver form | 3.732E-01 | 9.130E-03 |
| 323 | ALBU2 | Serum albumin 2 | 3.734E-01 | 6.736E-02 |
| 324 | ANT3 | Antithrombin-III | 3.734E-01 | 2.654E-02 |
| 325 | CD68 | Macrosialin | 3.736E-01 | 6.780E-02 |
| 326 | ALDR | Aldose reductase | 3.739E-01 | 6.461E-03 |
| 327 | ITIH2 | Inter-alpha-trypsin inhibitor heavy chain H2 | 3.748E-01 | 1.105E-02 |
| 328 | PLPL9 | 85/88 kDa calcium-independent phospholipase A2 | 3.762E-01 | 6.793E-02 |
| 329 | DHE3 | Glutamate dehydrogenase, mitochondrial | 3.786E-01 | 2.992E-02 |
| 330 | NFASC | Neurofascin | 3.804E-01 | 6.297E-02 |
| 331 | ALBU2 | Serum albumin 2 | 3.809E-01 | 3.068E-02 |
| 332 | G6PD | Glucose-6-phosphate 1-dehydrogenase | 3.819E-01 | 2.870E-02 |
| 333 | GAT6A | GATA-binding factor 6-A | 3.835E-01 | 3.634E-02 |
| 334 | C0HA95 | Calpastatin | 3.838E-01 | 4.251E-02 |
| 335 | PFD4 | Prefoldin subunit 4 | 3.838E-01 | 1.829E-02 |
| 336 | INP5K | Inositol polyphosphate 5-phosphatase K | 3.851E-01 | 2.479E-02 |
| 337 | A1AT | Alpha-1-antitrypsin | 3.851E-01 | 4.468E-02 |
| 338 | MACD1 | O-acetyl-ADP-ribose deacetylase MACROD1 | 3.854E-01 | 1.724E-02 |
| 339 | ZN503 | Zinc finger protein 503 | 3.860E-01 | 6.753E-02 |
| 340 | MYH11 | Myosin-11 | 3.863E-01 | 6.877E-02 |
| 341 | ZO61 | Oocyte zinc finger protein XlCOF6.1 | 3.865E-01 | 2.351E-02 |
| 342 | NUCB2 | Nesfatin-1 | 3.867E-01 | 3.102E-02 |
| 343 | NUCB2 | Nesfatin-1 | 3.867E-01 | 2.147E-02 |
| 344 | AP1M2 | AP-1 complex subunit mu-2 | 3.867E-01 | 2.584E-04 |
| 345 | Q5C6B2 | SJCHGC04011 protein | 3.892E-01 | 6.736E-02 |
| 346 | APOB | Apolipoprotein B-100 | 3.895E-01 | 5.533E-02 |
| 347 | ITAV | Integrin alpha-V light chain | 3.912E-01 | 2.479E-02 |
| 348 | NCPR | NADPH--cytochrome P450 reductase | 3.915E-01 | 5.922E-02 |
| 349 | ALBU1 | Serum albumin 1 | 3.917E-01 | 3.703E-02 |
| 350 | SURF4 | Surfeit locus protein 4 | 3.918E-01 | 1.602E-02 |
| 351 | MYH11 | Myosin-11 | 3.921E-01 | 6.439E-02 |
| 352 | FETUB | Fetuin-B | 3.932E-01 | 5.688E-02 |
| 353 | NUCB2 | Nesfatin-1 | 3.933E-01 | 4.691E-02 |
| 354 | EAF1 | ELL-associated factor 1 | 3.938E-01 | 7.107E-02 |
| 355 | ITA6 | Integrin alpha-6 | 3.945E-01 | 1.761E-02 |
| 356 | GIMA8 | GTPase IMAP family member 8 | 3.954E-01 | 2.147E-02 |
| 357 | PYGM | Glycogen phosphorylase, muscle form | 3.960E-01 | 5.044E-02 |
| 358 | S47A1 | Multidrug and toxin extrusion protein 1 | 3.961E-01 | 1.415E-02 |
| 359 | ALBU1 | Serum albumin 1 | 3.968E-01 | 4.020E-02 |
| 360 | CO9 | Complement component C9 | 3.969E-01 | 3.551E-02 |
| 361 | IC1 | Plasma protease C1 inhibitor | 3.972E-01 | 2.870E-02 |
| 362 | ANT3 | Antithrombin-III | 3.986E-01 | 1.688E-02 |
| 363 | ITAV | Integrin alpha-V light chain | 3.994E-01 | 4.020E-02 |
| 364 | ALBU2 | Serum albumin 2 | 3.996E-01 | 1.724E-02 |
| 365 | ALBU2 | Serum albumin 2 | 4.000E-01 | 1.161E-02 |
| 366 | ALBU2 | Serum albumin 2 | 4.001E-01 | 3.524E-02 |
| 367 | STRA6 | Stimulated by retinoic acid gene 6 protein homolog | 4.002E-01 | 5.075E-02 |
| 368 | GLPK5 | Putative glycerol kinase 5 | 4.003E-01 | 3.310E-02 |
| 369 | LSR | Lipolysis-stimulated lipoprotein receptor | 4.006E-01 | 3.717E-02 |
| 370 | FETUB | Fetuin-B | 4.007E-01 | 5.688E-02 |
| 371 | AMBP | Alpha-1-microglobulin | 4.026E-01 | 3.689E-02 |
| 372 | CG026 | Uncharacterized protein C7orf26 | 4.026E-01 | 2.968E-02 |
| 373 | APOB | Apolipoprotein B-100 | 4.027E-01 | 4.513E-02 |
| 374 | ESRP2 | Epithelial splicing regulatory protein 2 | 4.031E-01 | 6.780E-02 |
| 375 | I3K9D2 | Uncharacterized protein | 4.032E-01 | 6.831E-02 |
| 376 | QSOX1 | Sulfhydryl oxidase 1 | 4.035E-01 | 4.481E-03 |
| 377 | I3JY77 | Uncharacterized protein | 4.038E-01 | 6.693E-02 |
| 378 | IYD1 | Iodotyrosine dehalogenase 1 | 4.040E-01 | 5.125E-02 |
| 379 | ALBU2 | Serum albumin 2 | 4.040E-01 | 3.144E-02 |
| 380 | TMPS6 | Transmembrane protease serine 6 | 4.041E-01 | 3.689E-02 |
| 381 | ALBU1 | Serum albumin 1 | 4.045E-01 | 3.882E-02 |
| 382 | GATL2 | GATS-like protein 2 | 4.060E-01 | 5.557E-02 |
| 383 | ELP1 | Elongator complex protein 1 | 4.061E-01 | 6.780E-02 |
| 384 | CRVP | Cysteine-rich venom protein pseudecin | 4.070E-01 | 7.090E-02 |
| 385 | FSD1L | FSD1-like protein | 4.076E-01 | 5.617E-02 |
| 386 | PROC | Vitamin K-dependent protein C heavy chain | 4.077E-01 | 3.974E-02 |
| 387 | ISL2A | Insulin gene enhancer protein ISL-2A | 4.082E-01 | 5.115E-02 |
| 388 | TCPH | T-complex protein 1 subunit eta | 4.088E-01 | 1.639E-02 |
| 389 | FETUB | Fetuin-B | 4.096E-01 | 5.249E-02 |
| 390 | CAP2 | Adenylyl cyclase-associated protein 2 | 4.100E-01 | 1.724E-02 |
| 391 | ADT1 | ADP/ATP translocase 1 | 4.121E-01 | 1.495E-02 |
| 392 | ALBU1 | Serum albumin 1 | 4.123E-01 | 2.391E-02 |
| 393 | ALBU1 | Serum albumin 1 | 4.141E-01 | 2.395E-02 |
| 394 | LFG1 | Protein lifeguard 1 | 4.144E-01 | 3.961E-02 |
| 395 | ALBU2 | Serum albumin 2 | 4.145E-01 | 1.176E-02 |
| 396 | PROZ | Vitamin K-dependent protein Z | 4.162E-01 | 4.881E-02 |
| 397 | AMBP | Alpha-1-microglobulin | 4.164E-01 | 3.484E-02 |
| 398 | ALBU2 | Serum albumin 2 | 4.167E-01 | 2.654E-02 |
| 399 | IC1 | Plasma protease C1 inhibitor | 4.169E-01 | 2.557E-02 |
| 400 | ANO5 | Anoctamin-5 | 4.184E-01 | 1.219E-02 |
| 401 | ALDR | Aldose reductase | 4.189E-01 | 7.284E-04 |
| 402 | TINAL | Tubulointerstitial nephritis antigen-like | 4.195E-01 | 3.689E-02 |
| 403 | NEC2 | Neuroendocrine convertase 2 | 4.205E-01 | 4.287E-02 |
| 404 | IWS1 | Protein IWS1 homolog | 4.208E-01 | 8.558E-03 |
| 405 | ABCD3 | ATP-binding cassette sub-family D member 3 | 4.212E-01 | 6.768E-02 |
| 406 | ANT3 | Antithrombin-III | 4.220E-01 | 1.495E-02 |
| 407 | CERU | Ceruloplasmin | 4.238E-01 | 4.881E-02 |
| 408 | ALBU2 | Serum albumin 2 | 4.240E-01 | 1.449E-02 |
| 409 | OSTA | Organic solute transporter subunit alpha | 4.245E-01 | 6.166E-03 |
| 410 | ENPP2 | Ectonucleotide pyrophosphatase/phosphodiesterase family member 2 | 4.246E-01 | 3.494E-02 |
| 411 | ANT3 | Antithrombin-III | 4.248E-01 | 2.499E-03 |
| 412 | FIBB | Fibrinopeptide B | 4.250E-01 | 6.974E-02 |
| 413 | LAP4B | Lysosomal-associated transmembrane protein 4B | 4.268E-01 | 5.452E-02 |
| 414 | OSBL6 | Oxysterol-binding protein-related protein 6 | 4.274E-01 | 6.679E-02 |
| 415 | NAF1 | H/ACA ribonucleoprotein complex non-core subunit NAF1 | 4.276E-01 | 6.740E-02 |
| 416 | SCLY | Selenocysteine lyase | 4.276E-01 | 1.198E-03 |
| 417 | IC1 | Plasma protease C1 inhibitor | 4.281E-01 | 1.415E-02 |
| 418 | G6PE | 6-phosphogluconolactonase | 4.285E-01 | 2.717E-02 |
| 419 | CRVP | Cysteine-rich venom protein pseudecin | 4.295E-01 | 4.234E-02 |
| 420 | PYGO2 | Pygopus homolog 2 | 4.296E-01 | 5.763E-02 |
| 421 | P5I11 | Tumor protein p53-inducible protein 11 | 4.299E-01 | 2.203E-02 |
| 422 | RM14 | 39S ribosomal protein L14, mitochondrial | 4.311E-01 | 1.505E-02 |
| 423 | FETUA | Alpha-2-HS-glycoprotein | 4.323E-01 | 4.411E-02 |
| 424 | BHE23 | Class E basic helix-loop-helix protein 23 | 4.352E-01 | 5.628E-02 |
| 425 | CP2D3 | Cytochrome P450 2D3 | 4.358E-01 | 3.374E-02 |
| 426 | MTP | Microsomal triglyceride transfer protein large subunit | 4.363E-01 | 3.347E-02 |
| 427 | I3K9D2 | Uncharacterized protein | 4.371E-01 | 5.159E-02 |
| 428 | CP2G1 | Cytochrome P450 2G1 | 4.375E-01 | 2.968E-02 |
| 429 | SEM3D | Semaphorin-3D | 4.381E-01 | 6.097E-02 |
| 430 | IC1 | Plasma protease C1 inhibitor | 4.381E-01 | 9.780E-03 |
| 431 | G6PE | 6-phosphogluconolactonase | 4.387E-01 | 3.173E-02 |
| 432 | CP070 | UPF0183 protein C16orf70 | 4.396E-01 | 3.233E-02 |
| 433 | RCOR3 | REST corepressor 3 | 4.416E-01 | 4.673E-02 |
| 434 | GIMA7 | GTPase IMAP family member 7 | 4.420E-01 | 5.697E-02 |
| 435 | NFIP1 | NEDD4 family-interacting protein 1 | 4.422E-01 | 7.995E-03 |
| 436 | IMDH2 | Inosine-5'-monophosphate dehydrogenase 2 | 4.434E-01 | 4.631E-02 |
| 437 | I3JY77 | Uncharacterized protein | 4.439E-01 | 2.020E-02 |
| 438 | IC1 | Plasma protease C1 inhibitor | 4.456E-01 | 1.303E-02 |
| 439 | WIBGB | Partner of Y14 and mago B | 4.465E-01 | 1.275E-02 |
| 440 | WDR59 | WD repeat-containing protein 59 | 4.481E-01 | 7.140E-02 |
| 441 | GPX3 | Glutathione peroxidase 3 | 4.489E-01 | 1.388E-02 |
| 442 | ABCBB | Bile salt export pump | 4.500E-01 | 4.673E-02 |
| 443 | ANT3 | Antithrombin-III | 4.500E-01 | 1.495E-03 |
| 444 | STXB | Stonustoxin subunit beta | 4.515E-01 | 1.229E-02 |
| 445 | F1QCH5 | Uncharacterized protein | 4.520E-01 | 2.271E-02 |
| 446 | KDM7A | Lysine-specific demethylase 7A | 4.524E-01 | 2.602E-02 |
| 447 | DS7CB | Dehydrogenase/reductase SDR family member 7C-B | 4.538E-01 | 4.020E-02 |
| 448 | ANT3 | Antithrombin-III | 4.548E-01 | 1.144E-03 |
| 449 | IC1 | Plasma protease C1 inhibitor | 4.593E-01 | 7.295E-03 |
| 450 | PYGB | Glycogen phosphorylase, brain form | 4.600E-01 | 1.415E-02 |
| 451 | CRVP | Cysteine-rich venom protein pseudecin | 4.601E-01 | 4.509E-02 |
| 452 | RPGFL | Rap guanine nucleotide exchange factor-like 1 | 4.623E-01 | 6.040E-02 |
| 453 | TNMD | Tenomodulin | 4.634E-01 | 5.032E-02 |
| 454 | F1REA9 | Uncharacterized protein | 4.644E-01 | 4.720E-02 |
| 455 | MYH11 | Myosin-11 | 4.653E-01 | 4.812E-02 |
| 456 | NUCB2 | Nesfatin-1 | 4.656E-01 | 1.219E-02 |
| 457 | T161A | Transmembrane protein 161A | 4.657E-01 | 6.264E-02 |
| 458 | ODPB | Pyruvate dehydrogenase E1 component subunit beta, mitochondrial | 4.660E-01 | 5.922E-02 |
| 459 | FETUA | Alpha-2-HS-glycoprotein | 4.660E-01 | 3.484E-02 |
| 460 | RAMP1 | Receptor activity-modifying protein 1 | 4.675E-01 | 6.271E-02 |
| 461 | BGAL | Beta-galactosidase | 4.681E-01 | 1.303E-02 |
| 462 | PROZ | Vitamin K-dependent protein Z | 4.686E-01 | 1.336E-03 |
| 463 | SUCHY | Succinate--hydroxymethylglutarate CoA-transferase | 4.692E-01 | 6.546E-02 |
| 464 | SI1L3 | Signal-induced proliferation-associated 1-like protein 3 | 4.709E-01 | 4.020E-02 |
| 465 | HIPL1 | HHIP-like protein 1 | 4.715E-01 | 9.777E-04 |
| 466 | UHRF2 | E3 ubiquitin-protein ligase UHRF2 | 4.721E-01 | 4.603E-02 |
| 467 | ALBU2 | Serum albumin 2 | 4.722E-01 | 7.638E-04 |
| 468 | FSD1L | FSD1-like protein | 4.723E-01 | 4.007E-02 |
| 469 | CAP2 | Adenylyl cyclase-associated protein 2 | 4.727E-01 | 4.268E-02 |
| 470 | KGP2 | cGMP-dependent protein kinase 2 | 4.735E-01 | 6.743E-02 |
| 471 | CLD10 | Claudin-10 | 4.756E-01 | 5.130E-02 |
| 472 | NAA25 | N-alpha-acetyltransferase 25, NatB auxiliary subunit | 4.761E-01 | 7.162E-02 |
| 473 | PPCT | Phosphatidylcholine transfer protein | 4.763E-01 | 4.593E-02 |
| 474 | FETUA | Alpha-2-HS-glycoprotein | 4.782E-01 | 2.882E-02 |
| 475 | H2LJ69 | Uncharacterized protein | 4.799E-01 | 5.677E-02 |
| 476 | C1R | Complement C1r subcomponent | 4.808E-01 | 1.492E-02 |
| 477 | NALP9 | NACHT, LRR and PYD domains-containing protein 9 | 4.812E-01 | 5.697E-02 |
| 478 | ZN503 | Zinc finger protein 503 | 4.813E-01 | 3.144E-02 |
| 479 | H2LLV1 | Uncharacterized protein | 4.820E-01 | 6.770E-02 |
| 480 | PLMN | Plasmin heavy chain A | 4.825E-01 | 9.880E-03 |
| 481 | PPCT | Phosphatidylcholine transfer protein | 4.839E-01 | 4.128E-02 |
| 482 | MED17 | Mediator of RNA polymerase II transcription subunit 17 | 4.856E-01 | 2.217E-02 |
| 483 | PPCT | Phosphatidylcholine transfer protein | 4.860E-01 | 3.792E-02 |
| 484 | CACO1 | Calcium-binding and coiled-coil domain-containing protein 1 | 4.915E-01 | 4.297E-02 |
| 485 | CRVP | Cysteine-rich venom protein pseudecin | 4.915E-01 | 2.717E-02 |
| 486 | TSN9 | Tetraspanin-9 | 4.920E-01 | 4.318E-02 |
| 487 | I3JGD8 | Uncharacterized protein | 4.931E-01 | 4.629E-02 |
| 488 | PLIN2 | Perilipin-2 | 4.935E-01 | 5.851E-02 |
| 489 | FIBB | Fibrinopeptide B | 4.938E-01 | 3.173E-02 |
| 490 | H2LLV1 | Uncharacterized protein | 4.941E-01 | 6.271E-02 |
| 491 | NFIP1 | NEDD4 family-interacting protein 1 | 4.946E-01 | 9.777E-04 |
| 492 | I3JY77 | Uncharacterized protein | 4.964E-01 | 1.415E-02 |
| 493 | APOB | Apolipoprotein B-100 | 4.974E-01 | 6.297E-02 |
| 494 | I3K9D2 | Uncharacterized protein | 4.978E-01 | 3.484E-02 |
| 495 | SCOT1 | Succinyl-CoA:3-ketoacid coenzyme A transferase 1, mitochondrial | 4.986E-01 | 7.887E-03 |
| 496 | PTPRB | Receptor-type tyrosine-protein phosphatase beta | 4.991E-01 | 5.674E-02 |
| 497 | APOB | Apolipoprotein B-100 | 4.995E-01 | 3.789E-02 |
| 498 | DHR12 | Dehydrogenase/reductase SDR family member 12 | 5.020E-01 | 1.669E-02 |
| 499 | S13A2 | Solute carrier family 13 member 2 | 5.023E-01 | 7.004E-02 |
| 500 | P5I11 | Tumor protein p53-inducible protein 11 | 5.026E-01 | 6.097E-02 |
| 501 | DEN4C | DENN domain-containing protein 4C | 5.040E-01 | 4.979E-02 |
| 502 | NF2IP | NFATC2-interacting protein | 5.056E-01 | 2.968E-02 |
| 503 | A2AP | Alpha-2-antiplasmin | 5.072E-01 | 2.479E-02 |
| 504 | C1R | Complement C1r subcomponent | 5.074E-01 | 8.052E-03 |
| 505 | RB11A | Ras-related protein Rab-11A | 5.075E-01 | 6.439E-02 |
| 506 | ABCBB | Bile salt export pump | 5.079E-01 | 1.415E-02 |
| 507 | MED28 | Mediator of RNA polymerase II transcription subunit 28 | 5.111E-01 | 3.009E-02 |
| 508 | LBP | Lipopolysaccharide-binding protein | 5.141E-01 | 2.621E-02 |
| 509 | GUC1A | Guanylyl cyclase-activating protein 1 | 5.146E-01 | 1.761E-02 |
| 510 | LMBRL | Limb region 1 homolog-like protein | 5.154E-01 | 4.185E-02 |
| 511 | ABCBB | Bile salt export pump | 5.173E-01 | 2.430E-02 |
| 512 | PLMN | Plasmin heavy chain A | 5.175E-01 | 4.481E-03 |
| 513 | SEM7A | Semaphorin-7A | 5.179E-01 | 1.602E-02 |
| 514 | ANKH1 | Ankyrin repeat and KH domain-containing protein 1 | 5.200E-01 | 3.524E-02 |
| 515 | DJC21 | DnaJ homolog subfamily C member 21 | 5.266E-01 | 1.341E-02 |
| 516 | UBL3 | Ubiquitin-like protein 3 | 5.272E-01 | 3.108E-02 |
| 517 | CRVP | Cysteine-rich venom protein pseudecin | 5.275E-01 | 3.130E-02 |
| 518 | CBPB2 | Carboxypeptidase B2 | 5.289E-01 | 2.224E-02 |
| 519 | CEAM1 | Carcinoembryonic antigen-related cell adhesion molecule 1 | 5.296E-01 | 5.726E-02 |
| 520 | VA0D1 | V-type proton ATPase subunit d 1 | 5.296E-01 | 1.037E-02 |
| 521 | PEPA | Pepsin A | 5.319E-01 | 6.297E-02 |
| 522 | TM1L2 | TOM1-like protein 2 | 5.321E-01 | 5.591E-02 |
| 523 | DCTN4 | Dynactin subunit 4 | 5.322E-01 | 6.770E-02 |
| 524 | BNIP2 | BCL2/adenovirus E1B 19 kDa protein-interacting protein 2 | 5.337E-01 | 6.780E-02 |
| 525 | CBPB2 | Carboxypeptidase B2 | 5.340E-01 | 3.484E-02 |
| 526 | AL8A1 | Aldehyde dehydrogenase family 8 member A1 | 5.347E-01 | 3.484E-02 |
| 527 | IF5A1 | Eukaryotic translation initiation factor 5A-1 | 5.349E-01 | 4.007E-02 |
| 528 | ATS13 | A disintegrin and metalloproteinase with thrombospondin motifs 13 | 5.358E-01 | 5.283E-03 |
| 529 | APOB | Apolipoprotein B-100 | 5.371E-01 | 2.715E-02 |
| 530 | G3Q547 | Uncharacterized protein | 5.391E-01 | 4.691E-02 |
| 531 | IBP1 | Insulin-like growth factor-binding protein 1 | 5.397E-01 | 2.305E-02 |
| 532 | STXB | Stonustoxin subunit beta | 5.401E-01 | 3.703E-02 |
| 533 | C1R | Complement C1r subcomponent | 5.408E-01 | 8.161E-03 |
| 534 | HPHL1 | Hephaestin-like protein 1 | 5.421E-01 | 6.921E-02 |
| 535 | IGF2 | Insulin-like growth factor II | 5.433E-01 | 2.296E-02 |
| 536 | NB5R2 | NADH-cytochrome b5 reductase 2 | 5.443E-01 | 2.442E-02 |
| 537 | INSI2 | Insulin-induced gene 2 protein | 5.446E-01 | 1.388E-02 |
| 538 | IF2B3 | Insulin-like growth factor 2 mRNA-binding protein 3 | 5.446E-01 | 2.025E-02 |
| 539 | ISL1 | Insulin gene enhancer protein isl-1 | 5.447E-01 | 1.664E-02 |
| 540 | FETUB | Fetuin-B | 5.457E-01 | 5.127E-02 |
| 541 | IFT74 | Intraflagellar transport protein 74 homolog | 5.468E-01 | 4.296E-02 |
| 542 | CRVP | Cysteine-rich venom protein pseudecin | 5.480E-01 | 1.560E-02 |
| 543 | EGR2B | Early growth response protein 2b | 5.504E-01 | 1.616E-02 |
| 544 | LMA2L | VIP36-like protein | 5.509E-01 | 1.922E-02 |
| 545 | EKI1 | Ethanolamine kinase 1 | 5.510E-01 | 4.918E-02 |
| 546 | MYP0 | Myelin protein P0 | 5.511E-01 | 1.578E-02 |
| 547 | NLRC3 | Protein NLRC3 | 5.511E-01 | 2.180E-03 |
| 548 | FIBA | Fibrinopeptide A | 5.520E-01 | 1.219E-02 |
| 549 | C1R | Complement C1r subcomponent | 5.555E-01 | 1.279E-02 |
| 550 | TERA | Transitional endoplasmic reticulum ATPase | 5.558E-01 | 5.293E-02 |
| 551 | ACTN2 | Alpha-actinin-2 | 5.578E-01 | 5.260E-02 |
| 552 | TTPA | Alpha-tocopherol transfer protein | 5.588E-01 | 4.297E-02 |
| 553 | H2LLV1 | Uncharacterized protein | 5.624E-01 | 1.005E-02 |
| 554 | CEAM5 | Carcinoembryonic antigen-related cell adhesion molecule 5 | 5.624E-01 | 3.638E-02 |
| 555 | CRVP | Cysteine-rich venom protein pseudecin | 5.635E-01 | 1.166E-02 |
| 556 | C1R | Complement C1r subcomponent | 5.666E-01 | 7.995E-03 |
| 557 | PEAM3 | Phosphoethanolamine N-methyltransferase 3 | 5.687E-01 | 2.160E-03 |
| 558 | PFD4 | Prefoldin subunit 4 | 5.712E-01 | 1.670E-05 |
| 559 | IBP2B | Insulin-like growth factor-binding protein 2-B | 5.736E-01 | 2.442E-02 |
| 560 | INSI1 | Insulin-induced gene 1 protein | 5.768E-01 | 1.250E-02 |
| 561 | TRI16 | Tripartite motif-containing protein 16 | 5.814E-01 | 3.160E-02 |
| 562 | VTNC | Vitronectin | 5.829E-01 | 1.616E-02 |
| 563 | INSR | Insulin receptor subunit beta | 5.851E-01 | 9.780E-03 |
| 564 | A2AP | Alpha-2-antiplasmin | 5.853E-01 | 2.302E-02 |
| 565 | ITB4 | Integrin beta-4 | 5.864E-01 | 9.809E-03 |
| 566 | BT2A1 | Butyrophilin subfamily 2 member A1 | 5.867E-01 | 4.919E-02 |
| 567 | HPHL1 | Hephaestin-like protein 1 | 5.873E-01 | 5.217E-02 |
| 568 | IBP2A | Insulin-like growth factor-binding protein 2-A | 5.885E-01 | 2.442E-02 |
| 569 | ST3A1 | Amine sulfotransferase | 5.897E-01 | 4.067E-02 |
| 570 | FZD4 | Frizzled-4 | 5.906E-01 | 3.484E-02 |
| 571 | FZD6 | Frizzled-6 | 5.914E-01 | 4.012E-02 |
| 572 | G3N629 | Uncharacterized protein | 5.928E-01 | 4.604E-02 |
| 573 | SC6A1 | Sodium- and chloride-dependent GABA transporter 1 | 5.941E-01 | 2.319E-02 |
| 574 | PKNX1 | Homeobox protein PKNOX1 | 5.945E-01 | 6.919E-02 |
| 575 | METH | Methionine synthase | 5.958E-01 | 1.264E-02 |
| 576 | B3AT | Band 3 anion exchange protein | 5.965E-01 | 1.388E-02 |
| 577 | IBP3 | Insulin-like growth factor-binding protein 3 | 5.970E-01 | 2.450E-02 |
| 578 | G3Q5W3 | Uncharacterized protein | 5.976E-01 | 5.387E-02 |
| 579 | IDE | Insulin-degrading enzyme | 6.000E-01 | 1.079E-02 |
| 580 | ZN143 | Zinc finger protein 143 | 6.016E-01 | 6.439E-02 |
| 581 | FIBP | Acidic fibroblast growth factor intracellular-binding protein | 6.018E-01 | 2.323E-02 |
| 582 | YQK1 | UPF0676 protein C1494.01 | 6.039E-01 | 6.370E-05 |
| 583 | MMUM | Homocysteine S-methyltransferase | 6.083E-01 | 3.752E-02 |
| 584 | WNT2B | Protein Wnt-2b | 6.091E-01 | 6.036E-02 |
| 585 | PUA1B | Adenylosuccinate synthetase isozyme 1 B | 6.092E-01 | 6.163E-03 |
| 586 | CK5P3 | CDK5 regulatory subunit-associated protein 3 | 6.110E-01 | 3.108E-02 |
| 587 | IF2B2 | Insulin-like growth factor 2 mRNA-binding protein 2 | 6.110E-01 | 1.829E-02 |
| 588 | IRS2 | Insulin receptor substrate 2 | 6.117E-01 | 4.590E-03 |
| 589 | ACTN3 | Alpha-actinin-3 | 6.133E-01 | 2.870E-02 |
| 590 | C1QBP | Complement component 1 Q subcomponent-binding protein, mitochondrial | 6.139E-01 | 2.801E-02 |
| 591 | ACAC | Biotin carboxylase | 6.144E-01 | 7.126E-02 |
| 592 | COT2 | COUP transcription factor 2 | 6.155E-01 | 2.147E-02 |
| 593 | ARH | Low density lipoprotein receptor adapter protein 1 | 6.160E-01 | 3.789E-02 |
| 594 | MFD4A | Major facilitator superfamily domain-containing protein 4-A | 6.164E-01 | 5.383E-02 |
| 595 | G3P | Glyceraldehyde-3-phosphate dehydrogenase | 6.169E-01 | 2.305E-02 |
| 596 | AKAP1 | A-kinase anchor protein 1, mitochondrial | 6.172E-01 | 1.485E-03 |
| 597 | CD3Z | T-cell surface glycoprotein CD3 zeta chain | 6.213E-01 | 2.597E-02 |
| 598 | SNTB1 | Beta-1-syntrophin | 6.245E-01 | 1.037E-02 |
| 599 | FRRS1 | Ferric-chelate reductase 1 | 6.264E-01 | 4.817E-02 |
| 600 | FETUB | Fetuin-B | 6.264E-01 | 1.388E-02 |
| 601 | IGF1 | Insulin-like growth factor I | 6.288E-01 | 2.203E-02 |
| 602 | CELF2 | CUGBP Elav-like family member 2 | 6.343E-01 | 3.144E-02 |
| 603 | B3DFX3 | Hypothetical LOC561161 | 6.347E-01 | 3.045E-02 |
| 604 | ARH | Low density lipoprotein receptor adapter protein 1 | 6.364E-01 | 6.736E-02 |
| 605 | HINT2 | Histidine triad nucleotide-binding protein 2, mitochondrial | 6.386E-01 | 4.864E-02 |
| 606 | ADXL | Adrenodoxin-like protein, mitochondrial | 6.393E-01 | 1.303E-02 |
| 607 | PDZ11 | PDZ domain-containing protein 11 | 6.403E-01 | 3.108E-02 |
| 608 | LENG8 | Leukocyte receptor cluster member 8 homolog | 6.408E-01 | 2.610E-02 |
| 609 | WBP2 | WW domain-binding protein 2 | 6.409E-01 | 4.507E-02 |
| 610 | RPAB5 | DNA-directed RNA polymerases I, II, and III subunit RPABC5 | 6.425E-01 | 1.724E-02 |
| 611 | CL17A | C-type lectin domain family 17, member A | 6.430E-01 | 2.113E-02 |
| 612 | ABHD6 | Monoacylglycerol lipase ABHD6 | 6.440E-01 | 3.524E-02 |
| 613 | IF2B1 | Insulin-like growth factor 2 mRNA-binding protein 1 | 6.453E-01 | 1.388E-02 |
| 614 | DHI2 | Corticosteroid 11-beta-dehydrogenase isozyme 2 | 6.458E-01 | 1.649E-02 |
| 615 | INSR | Insulin receptor subunit alpha | 6.469E-01 | 8.979E-03 |
| 616 | IRS2A | Insulin receptor substrate 2-A | 6.469E-01 | 5.668E-03 |
| 617 | I3JLT0 | Uncharacterized protein | 6.477E-01 | 6.999E-03 |
| 618 | G3PD89 | Uncharacterized protein | 6.491E-01 | 6.423E-02 |
| 619 | IRS2B | Insulin receptor substrate 2-B | 6.494E-01 | 8.504E-03 |
| 620 | FEM1C | Protein fem-1 homolog C | 6.505E-01 | 1.279E-02 |
| 621 | CATZ | Cathepsin Z | 6.508E-01 | 2.968E-02 |
| 622 | ELOA1 | Transcription elongation factor B polypeptide 3 | 6.516E-01 | 6.864E-02 |
| 623 | FBXW7 | F-box/WD repeat-containing protein 7 | 6.528E-01 | 5.674E-02 |
| 624 | Q5DG26 | SJCHGC04882 protein | 6.536E-01 | 1.526E-02 |
| 625 | ABHD6 | Monoacylglycerol lipase ABHD6 | 6.550E-01 | 1.388E-02 |
| 626 | P2R3B | Serine/threonine-protein phosphatase 2A regulatory subunit B'' subunit beta | 6.583E-01 | 1.045E-02 |
| 627 | CO3 | Complement C3d fragment | 6.587E-01 | 4.211E-02 |
| 628 | MCHR1 | Melanin-concentrating hormone receptor 1 | 6.628E-01 | 1.236E-02 |
| 629 | TERA | Transitional endoplasmic reticulum ATPase | 6.645E-01 | 4.812E-02 |
| 630 | SAMH1 | Deoxynucleoside triphosphate triphosphohydrolase SAMHD1 | 6.647E-01 | 1.922E-02 |
| 631 | S27A6 | Long-chain fatty acid transport protein 6 | 6.670E-01 | 6.370E-05 |
| 632 | SOSSC | SOSS complex subunit C | 6.672E-01 | 6.576E-02 |
| 633 | Q5BWU0 | SJCHGC07585 protein | 6.681E-01 | 1.037E-02 |
| 634 | IRS1 | Insulin receptor substrate 1 | 6.708E-01 | 2.945E-03 |
| 635 | TMTC3 | Transmembrane and TPR repeat-containing protein 3 | 6.754E-01 | 2.395E-02 |
| 636 | AL8A1 | Aldehyde dehydrogenase family 8 member A1 | 6.760E-01 | 6.461E-03 |
| 637 | ANGL3 | Angiopoietin-related protein 3 | 6.763E-01 | 3.934E-03 |
| 638 | HSPB7 | Heat shock protein beta-7 | 6.769E-01 | 6.316E-02 |
| 639 | ANPRA | Atrial natriuretic peptide receptor 1 | 6.789E-01 | 1.219E-02 |
| 640 | METK1 | S-adenosylmethionine synthase isoform type-1 | 6.801E-01 | 6.200E-02 |
| 641 | ACAC | Biotin carboxylase | 6.815E-01 | 1.740E-02 |
| 642 | PDIA3 | Protein disulfide-isomerase A3 | 6.820E-01 | 5.013E-03 |
| 643 | CO3 | Complement C3d fragment | 6.835E-01 | 2.168E-02 |
| 644 | IBP4 | Insulin-like growth factor-binding protein 4 | 6.879E-01 | 5.628E-03 |
| 645 | COL12 | Collectin-12 | 6.882E-01 | 4.731E-02 |
| 646 | K121L | Uncharacterized protein KIAA1211-like | 6.892E-01 | 3.149E-02 |
| 647 | CTTB2 | Cortactin-binding protein 2 | 6.904E-01 | 7.160E-03 |
| 648 | ABHD6 | Monoacylglycerol lipase ABHD6 | 6.923E-01 | 1.669E-02 |
| 649 | BC7BB | B-cell CLL/lymphoma 7 protein family member B-B | 6.924E-01 | 1.562E-02 |
| 650 | STYK1 | Tyrosine-protein kinase STYK1 | 6.937E-01 | 2.452E-02 |
| 651 | CRHBP | Corticotropin-releasing factor-binding protein | 6.958E-01 | 2.020E-02 |
| 652 | GNAI1 | Guanine nucleotide-binding protein G(i) subunit alpha-1 | 6.964E-01 | 7.915E-03 |
| 653 | SAMH1 | Deoxynucleoside triphosphate triphosphohydrolase SAMHD1 | 6.974E-01 | 5.307E-02 |
| 654 | C0HA75 | Transposable element Tcb2 transposase | 7.000E-01 | 5.743E-02 |
| 655 | CO3 | C3a anaphylatoxin | 7.025E-01 | 7.490E-03 |
| 656 | SYTL1 | Synaptotagmin-like protein 1 | 7.035E-01 | 1.100E-02 |
| 657 | CFAB | Complement factor B Bb fragment | 7.057E-01 | 3.374E-02 |
| 658 | IBP5 | Insulin-like growth factor-binding protein 5 | 7.146E-01 | 6.662E-03 |
| 659 | D3GN50 | Defensin beta 3 | 7.157E-01 | 2.180E-03 |
| 660 | FA9 | Coagulation factor IXa heavy chain | 7.209E-01 | 1.587E-03 |
| 661 | Q5TZC8 | Uncharacterized protein | 7.274E-01 | 3.600E-02 |
| 662 | ABHD6 | Monoacylglycerol lipase ABHD6 | 7.295E-01 | 6.538E-03 |
| 663 | ARH | Low density lipoprotein receptor adapter protein 1 | 7.299E-01 | 1.761E-02 |
| 664 | GRN | Granulin-2 | 7.306E-01 | 4.825E-03 |
| 665 | YB039 | Uncharacterized protein LINC00116 homolog | 7.327E-01 | 2.602E-02 |
| 666 | ASB10 | Ankyrin repeat and SOCS box protein 10 | 7.412E-01 | 2.120E-02 |
| 667 | METH | Methionine synthase | 7.417E-01 | 2.180E-03 |
| 668 | YQK1 | UPF0676 protein C1494.01 | 7.419E-01 | 1.670E-05 |
| 669 | CFAB | Complement factor B | 7.436E-01 | 1.336E-03 |
| 670 | RHBG | Ammonium transporter Rh type B | 7.473E-01 | 4.600E-05 |
| 671 | CNBP1 | Beta-catenin-interacting protein 1 | 7.484E-01 | 5.117E-03 |
| 672 | UBL3 | Ubiquitin-like protein 3 | 7.493E-01 | 1.639E-02 |
| 673 | A3KNV7 | Si:dkeyp-35b8.5 protein | 7.503E-01 | 5.013E-03 |
| 674 | SQRD | Sulfide:quinone oxidoreductase, mitochondrial | 7.513E-01 | 3.689E-02 |
| 675 | G3PGC4 | Uncharacterized protein | 7.543E-01 | 1.740E-02 |
| 676 | SASB | Fatty acyl-CoA hydrolase precursor, medium chain | 7.562E-01 | 1.724E-02 |
| 677 | SUCO | SUN domain-containing ossification factor | 7.648E-01 | 2.471E-02 |
| 678 | CHIP | STIP1 homology and U box-containing protein 1 | 7.648E-01 | 6.461E-03 |
| 679 | Q9W709 | Transposase | 7.707E-01 | 4.398E-02 |
| 680 | FRRS1 | Ferric-chelate reductase 1 | 7.710E-01 | 1.761E-02 |
| 681 | Q5C6B2 | SJCHGC04011 protein | 7.850E-01 | 3.582E-04 |
| 682 | STXB | Stonustoxin subunit beta | 7.860E-01 | 2.207E-02 |
| 683 | CO3 | Complement C3b alpha' chain | 7.897E-01 | 1.619E-03 |
| 684 | COL12 | Collectin-12 | 8.029E-01 | 1.082E-03 |
| 685 | CLCN3 | H(+)/Cl(-) exchange transporter 3 | 8.080E-01 | 6.607E-02 |
| 686 | ALBU2 | Serum albumin 2 | 8.095E-01 | 2.077E-02 |
| 687 | SQRD | Sulfide:quinone oxidoreductase, mitochondrial | 8.178E-01 | 7.313E-03 |
| 688 | C0HA75 | Transposable element Tcb2 transposase | 8.202E-01 | 2.717E-02 |
| 689 | DHC24 | Delta(24)-sterol reductase | 8.284E-01 | 2.397E-02 |
| 690 | TC1A | Transposable element Tc1 transposase | 8.317E-01 | 5.260E-02 |
| 691 | CLC4E | C-type lectin domain family 4 member E | 8.344E-01 | 3.428E-02 |
| 692 | S27A6 | Long-chain fatty acid transport protein 6 | 8.413E-01 | 6.000E-05 |
| 693 | OVOS2 | Ovostatin homolog 2 | 8.427E-01 | 2.026E-02 |
| 694 | HIUH | 5-hydroxyisourate hydrolase | 8.493E-01 | 4.278E-02 |
| 695 | CASZ1 | Zinc finger protein castor homolog 1 | 8.532E-01 | 3.276E-02 |
| 696 | Q6ZVM5 | cDNA FLJ42083 fis, clone TCERX2000613 | 8.663E-01 | 5.851E-02 |
| 697 | CL17A | C-type lectin domain family 17, member A | 8.690E-01 | 1.168E-04 |
| 698 | ALBU2 | Serum albumin 2 | 8.694E-01 | 1.587E-03 |
| 699 | AL8A1 | Aldehyde dehydrogenase family 8 member A1 | 8.732E-01 | 4.790E-05 |
| 700 | NR0B2 | Nuclear receptor subfamily 0 group B member 2 | 8.782E-01 | 6.877E-02 |
| 701 | I3K7D2 | Uncharacterized protein | 8.866E-01 | 3.786E-02 |
| 702 | I3J6L6 | Uncharacterized protein | 8.951E-01 | 1.309E-02 |
| 703 | ALBU2 | Serum albumin 2 | 9.089E-01 | 9.785E-04 |
| 704 | CISH | Cytokine-inducible SH2-containing protein | 9.093E-01 | 3.879E-02 |
| 705 | S27A6 | Long-chain fatty acid transport protein 6 | 9.099E-01 | 1.400E-03 |
| 706 | TMED6 | Transmembrane emp24 domain-containing protein 6 | 9.107E-01 | 9.960E-05 |
| 707 | ALBU1 | Serum albumin 1 | 9.399E-01 | 2.505E-04 |
| 708 | I3L019 | Uncharacterized protein | 9.546E-01 | 6.297E-02 |
| 709 | ALBU2 | Serum albumin 2 | 1.000E+00 | 3.780E-05 |
| 710 | I3JIB6 | Uncharacterized protein | 1.007E+00 | 1.600E-03 |
| 711 | SMRD3 | SWI/SNF-related matrix-associated actin-dependent regulator of chromatin subfamily D member 3 | 1.024E+00 | 1.958E-02 |
| 712 | CAP2 | Adenylyl cyclase-associated protein 2 | 1.110E+00 | 3.009E-02 |
| 713 | ITIH3 | Inter-alpha-trypsin inhibitor heavy chain H3 | 1.213E+00 | 6.780E-02 |
| 714 | ITIH3 | Inter-alpha-trypsin inhibitor heavy chain H3 | 1.218E+00 | 5.382E-02 |
| 715 | UD2A3 | UDP-glucuronosyltransferase 2A3 | 1.257E+00 | 2.054E-02 |
| 716 | COX1 | Cytochrome c oxidase subunit 1 | 1.269E+00 | 6.012E-02 |
| 717 | STXB | Stonustoxin subunit beta | 1.383E+00 | 5.677E-02 |
| 718 | OPN5 | Opsin-5 | 1.388E+00 | 2.748E-02 |
| 719 | ITIH3 | Inter-alpha-trypsin inhibitor heavy chain H3 | 1.467E+00 | 3.524E-02 |
| 720 | ITIH3 | Inter-alpha-trypsin inhibitor heavy chain H3 | 1.516E+00 | 5.755E-02 |
| 721 | GIMA5 | GTPase IMAP family member 5 | 3.869E+00 | 6.163E-03 |
| 722 | C1QL2 | Complement C1q-like protein 2 | 3.930E+00 | 1.641E-02 |
| 723 | R7TU90 | Uncharacterized protein | 4.462E+00 | 4.287E-04 |
